# Energy Transfer as A Driving Force in Nucleic Acid–Protein Interactions

**DOI:** 10.3390/molecules24071443

**Published:** 2019-04-11

**Authors:** Elena Zavyalova, Alexey Kopylov

**Affiliations:** Chemistry Department, Lomonosov Moscow State University, 119991 Moscow, Russia; kopylov.alex@gmail.com

**Keywords:** aptamer, protein, structure-activity relationship, energy dissipation, affinity

## Abstract

Many nucleic acid–protein structures have been resolved, though quantitative structure-activity relationship remains unclear in many cases. Thrombin complexes with G-quadruplex aptamers are striking examples of a lack of any correlation between affinity, interface organization, and other common parameters. Here, we tested the hypothesis that affinity of the aptamer–protein complex is determined with the capacity of the interface to dissipate energy of binding. Description and detailed analysis of 63 nucleic acid–protein structures discriminated peculiarities of high-affinity nucleic acid–protein complexes. The size of the amino acid sidechain in the interface was demonstrated to be the most significant parameter that correlates with affinity of aptamers. This observation could be explained in terms of need of efficient energy transfer from interacting residues. Application of energy dissipation theory provided an illustrative tool for estimation of efficiency of aptamer–protein complexes. These results are of great importance for a design of efficient aptamers.

## 1. Introduction

Proteins are macromolecules with a variety of functions in living cells. Protein functioning is supported by an ability to bind target specifically substances. Moreover, some proteins and enzymes catalyze further chemical conversion of bound substances. Several decades of intensive study of proteins have yielded large databases of structures of proteins and their complexes, thermodynamics of binding and catalysis, as well as kinetic data [1,2,3,4,5]. Many successful attempts to explain how structural features of the protein affect thermodynamic and kinetic parameters of interactions with a target have been reported [6,7,8,9,10,11]. However, to date, there is no general concept that allows prediction of affinity to the target or rate of enzymatic catalysis. Empirical and semi-empirical algorithms with parametrization of each interaction are widely used in docking [12,13,14], but the empiric component inevitably leads to a limited range of ligands that can be described with a good predictive force. These limitations obviously reflect overestimation of selected interactions and underestimation of some other significant aspects of protein function. Here, we describe a further attempt to find a clear and intelligible explanation of affinity of proteins to their ligands that would have not only descriptive nature but also a predictive ability.

A striking example of non-understanding of high affinity of nucleic acid–protein complexes has been reported recently [15]. We analyzed a set of complexes of thrombin with its artificial nucleic acid ligands, DNA aptamers. Different aptamers bind the same site of the thrombin having 100-fold different affinities. Moreover, there was no general correlation between aptamer affinity and parameters of the interface, such as interface area, number of atoms in the interface, and number of polar contacts. Even more, a detailed analysis of polar contacts of the best ligand and the worst one revealed no significant differences. Similarly, there was no correlation between thermodynamic parameters of aptamer structure and its affinity to thrombin. 

Expanding this specific dataset to all known nucleic acid aptamer–protein complexes, we also did not find a clear correlation between interface structure and aptamer affinity to the protein [16]. These results stimulated us to search for a reason for high affinity in kinetic processes during complex formation instead of just comparing the initial and final states of the molecules. We speculated that it is the energy released during the first steps of ligand binding that could unfold the interacting molecules destroying the intermediate complex before the final complex formation [15]. If this is the case, a dissipation of this binding energy from the interacting residues will enhance the rate of complex formation, and therefore increase the affinity. 

We made a further attempt to find a structure–affinity relationship for all nucleic acid–protein complexes available in the databases. The key processes that mediate energy transfer can be the following: (1) changes in H-bonds with water near the interface; (2) conformational rearrangements of the protein and the aptamer; and (3) redistribution of binding energy via residues with large sidechains that are located near the interface. In this work, amino acids from the interfaces have been thoroughly annotated and analyzed. 

## 2. Results

### 2.1. Complexes of Proteins with Nucleic Acid Aptamers

The dataset contained nucleic acid aptamer–protein complexes extracted from the Protein Data Bank [1] with the following criteria:

(1) X-ray structures have a resolution less than 3 Å;
(2) Only binary complexes (1 protein, 1 aptamer) were chosen to minimize allosteric effects;
(3) Apparent equilibrium constants for the complex formation are known.

Thirty-five complexes were analyzed, of which thirteen were with the same protein, human thrombin (Table 1). 

As for the nature of nucleic acid aptamers, DNA, RNA, modified DNA, and modified RNA aptamers were in the set. This set almost coincides with our previous work, where an explicit analysis of aptamer nature-affinity correlation was described [16]. Kinetic constants for complex association and dissociation were reported for several complexes and annotated in Table 1. Apparent equilibrium dissociation constants were recalculated into the changes in Gibbs free energy during binding using the equation ΔG_b_ = RT × lnK_d_, where R is gas constant, and T is temperature of the binding assay.

Several approaches were applied to analyze the interfaces of aptamer–protein complexes, having selected differences in amino acids. These include:

(1) Annotation of the amino acids that participate in polar contacts;
(2) Annotation of the amino acids located within 4 Å vicinity to atoms that participate in polar contacts;
(3) Annotation of the amino acids located within 4 Å vicinity to nucleotides that form 3 or more polar contacts (putative “hot spots”).

**Figure 1:**
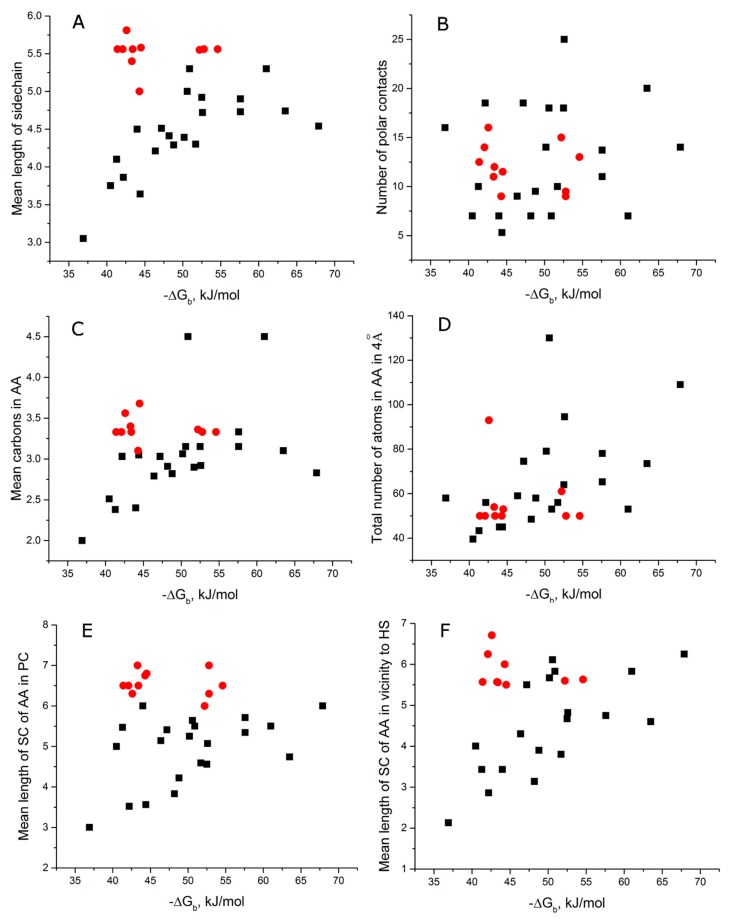
Aptamer–protein complexes. Dependencies of the mean length of the sidechain of amino acids within 4 Å proximity to polar contacts (**A**), number of polar contacts (**B**), mean number of carbon atoms in aromatic or aliphatic groups of the sidechain (**C**), and total number of atoms in amino acids within 4 Å proximity to polar contacts (**D**) versus change in Gibbs free energy during binding. The data are clustered—G-quadruplex aptamers with thrombin are colored in red, and all other aptamer complexes are colored in black.

All annotations are summarized in Table S1; derived values are summarized in Tables S2 and S3. As in our previous work [16], there was no correlation between changes in Gibbs free energy and other parameters for the whole dataset. However, when we split the dataset into a subset of G-quadruplex aptamers to thrombin and a subset with all others, the correlation became obvious (Table 2, Figure 1). 

Considering the total interface, i.e., the amino acids located within 4 Å vicinity to atoms that participate in polar contacts, G-quadruplex aptamers to thrombin formed a specific group that is located outside of diagonal distributions for other aptamers (Figure 1A). Analyzing all other aptamers revealed that the least dispersion of the heterogeneous dataset was for the mean length of sidechain (Table 2, Figure 1A). Mean length of sidechain is a characteristic of amino acid size and volume. It was calculated as a mean number of atoms in Cα substituents of amino acids, excluding hydrogen atoms (i.e., number of C, N, O, and S). Positive correlation between this parameter and −ΔG_b_ means that large sidechains are much more common in complexes with high affinity. This effect cannot been attributed solely to high amount of aromatic amino acids (“lengths” parameters are in the range 7–10) or positively charged amino acids (“lengths” parameters are in the range 5–7), as is seen from correlation coefficients < 0.2 (Table 2).

Diagonal distribution was characteristic for mean length parameters for other amino acid sets, namely, for amino acids making polar contacts (Figure 1E), amino acids within 4 Å vicinity to “hot spots” (Figure 1F), and mean number of aromatic or aliphatic carbons in amino acids in 4 Å vicinity of polar contacts (Figure 1C). Recurring correlation between –ΔG_b_ and mean length parameter supports the speculation that affinity increases when the binding energy from the reactive residues is dissipated. “Mean length” parameter is proportional to the mean volume of residues; the more residues that participate in energy distribution, the tighter the complex can be formed. The number of polar contacts and the total number of atoms had no correlation with −ΔG_b_ (Figure 1B,D), in agreement with previous observations for aptamer–protein complexes [15,16], and indicating that nucleic acid–protein complexes are more than an interaction between two complimentary surfaces.

**Figure 2:**
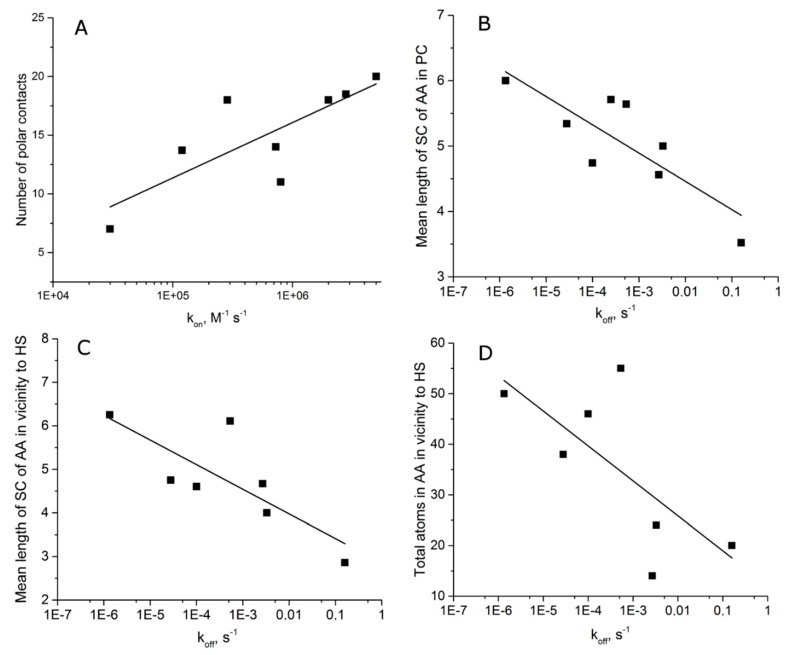
Analysis of kinetic parameters of aptamer–protein complexes. (**A**) The dependencies of number of polar contacts (PC) from kinetic constants of association, k_on_. (**B**) The mean length sidechain of amino-acid-formed PC from kinetic constant of dissociation, k_off_. (**C**) The mean length sidechain of amino acids within 4 Å proximity to “hot spots” (HS) versus k_off_. (**D**) Total number of atoms in amino acids within 4 Å proximity to HS versus, k_off_ (**D**). Logarithmic (y = a × ln(x) + b) approximations were used.

**Figure 3:**
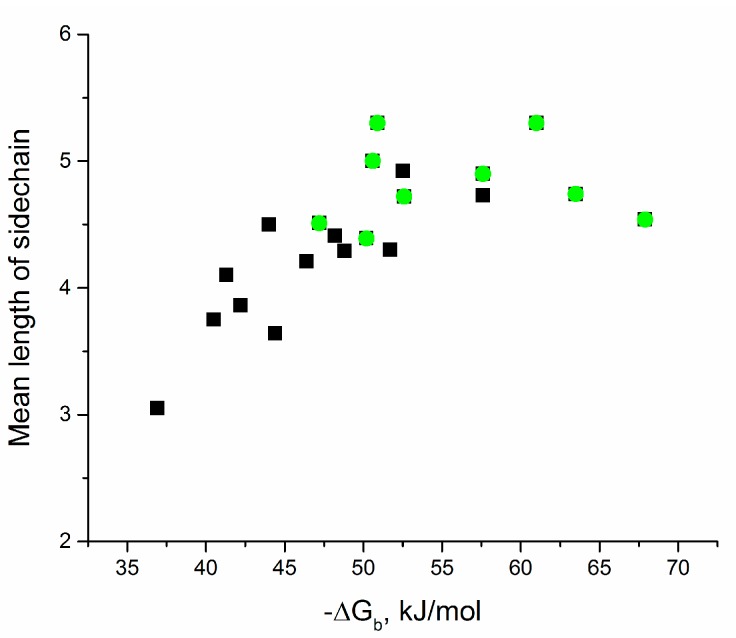
Selection of high-affinity complexes based on the parameters of the interface. Complexes with high values of the parameters of the number of polar contacts (PC), the mean length sidechain of amino-acid-formed PC and amino acids within 4 Å proximity to “hot spots” (HS), and the total number of atoms in amino acids within 4 Å proximity to HS (green dots) have the highest changes in Gibbs free energy during binding compared to other complexes (black dots).

Kinetic constants of association and dissociation were described for 8 of 21 complexes from this subset. We analyzed this small dataset in more detail. The results were quite interesting (Table 3, Figure 2). Kinetic constant of association is correlated with the number of polar contacts only, whereas kinetic constant of dissociation is correlated with mean length of sidechain of amino acids making polar contacts and amino acids within 4 Å vicinity of the putative “hot spot”. Thus, a large pattern of polar contacts provides fast complex formation, whereas the possibility to dissipate the binding energy from residues involved in these contacts supports the high stability of the complex. This suggestion was tested using the extended dataset of aptamers (Figure 3), where the complexes with the highest values of the above parameters were chosen (the parameters are the number of polar contacts, the mean length of sidechain of amino acids making polar contacts, the mean length of sidechain of amino acids within 4 Å vicinity of the putative “hot spot”, and the total number of atoms in amino acids in the 4 Å vicinity of “hot spots”). As a result, 8 from 11 aptamers with ΔG_b_ ≥ 50 kJ/mol met these criteria. Thus, for the first time the exact parameters of the interface that are critical for aptamer affinity were found and proved for the whole dataset. 

### 2.2. Complexes of HTH-type Proteins with DNA Double Helixes

An interesting and well-studied object is complexes of HTH-type proteins with DNA double helixes. HTH-type proteins have a specific DNA binding motif: helix–turn–helix (HTH). They are a classical object of studying DNA binding and recognition. Therefore, comparing interfaces of complexes of proteins with artificial nucleic acid aptamers and natural DNA double helices is of great value. Criteria for this dataset were the following:

(1) X-ray structures have a resolution less than 3 Å;
(2) X-ray structure is for the whole protein, not a protein domain;
(3) DNA has unmodified nucleotides only;
(4) Apparent equilibrium constants for the complex are known.

The selected set has bacterial proteins, including mesophiles and one thermophile (G. stearothermophilus) (Table 4). The size of proteins varied from 62 to 246 residues, and the typical size of the DNA duplex was about 25 base pairs. Kinetic constants for complex association and dissociation were reported for 2 complexes only. Apparent equilibrium dissociation constants were recalculated into changes in Gibbs free energy using the equation ΔG_b_ = RT lnK_d_, where R is gas constant, and T is temperature of the binding assay. For one of the proteins, fis, 18 complexes with different DNA duplexes were described, including optimal and non-optimal ones. All these complexes were analyzed in the same way as aptamer–protein complexes described above (Tables S4 and S5).

**Figure 4:**
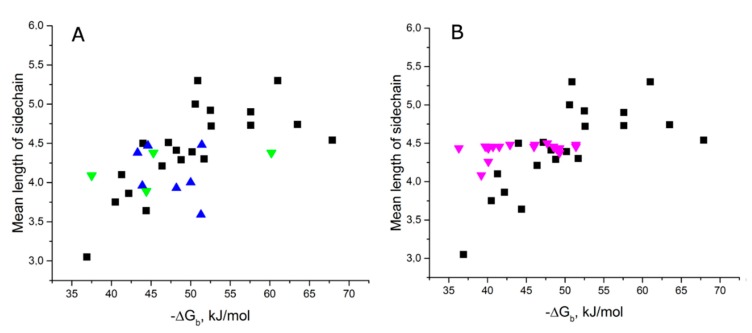
Comparison of aptamer–protein and DNA helix–protein complexes plotted in the coordinates mean lengths of sidechain of amino acids within 4 Å vicinity of polar contacts versus changes in Gibbs free energy during binding. (**A**) HTH-type proteins from mesophiles with human-like conditions (blue dots), and other organisms (Bacillus and a thermophile = green dots) were plotted with aptamer–protein complexes (black dots). (**B**) Complexes of fis protein with optimal and non-optimal DNA helices (magenta dots) were plotted with aptamer–protein complexes (black dots).

The datasets of aptamer–protein and DNA helix–protein complexes have similar distributions, e.g., mean length of sidechain of amino acids within 4 Å vicinity of polar contacts versus −ΔG_b_ (Figure 4A). This similarity reflects similar organization of the interfaces, but HTH-type proteins had no obvious diagonal distribution per se (Figure 4A). Interesting results were obtained from analyzing optimal and non-optimal complexes of fis protein with different DNA duplexes (Figure 4B). These complexes have very similar interfaces, but drastically different apparent dissociation constants in the range from 0.2 nM to 140 nM. The tightest complexes are located on the diagonal distribution of aptamer–protein complexes, whereas non-optimal complexes were on the left side of the distribution. Thus, diagonal distribution could be used as a measure of efficiency of complex formation.

## 3. Discussion

In contemporary conception, water as a solvent is the most efficient receiver of the excessive energy during dissipation. The water arrangement of the protein is dynamic. It fluctuates due to thermal excitation of low-frequency modes, and hydrogen bonds are broken and reformed within roughly 1 ps [14]. As for protein complexes, a considerable part of the interface has no direct interactions with the solvent. Thus, the protein or its counterpart do participate in dissipation of energy from the polar contact-forming residues to solvent. 

In extreme cases, such as in plant and algae photosystems, there are special proteins that mediate efficient dissipation of energy from the light-harvesting complexes [41,42,43]. This additional help becomes critical under high light exposure. For this case, the typical time for dissipation of energy is around 20 ps (τ_1/2_) [41]. As for non-assisted dissipation of energy, in silico calculations gave typical time scales in the range from 10 ps to 10 ns for single proteins [44,45], and time for energy transfer to the nearby residue is about 0.5 ps [46]. Comparing the time scales, experimental techniques revealed conformational rearrangement of DNA oligonucleotide to proceed during 8 µs and fast steps of protein folding during 90 µs [47]. 

Direct experimental study of dissipation of energy in proteins without unusual prosthetic groups is complicated due to inability to trace specific residues only. However, bioinformatic analysis provided clues of a role of energy dissipation in protein functioning. A bridge between affinity and capacity for information transmission was paved for DNA–protein complexes [48,49]. The dissipated energy for a bit of transmitted information is defined with the equation:

(1) $$\varepsilon =\frac{P_{y}}{C_{y}}$$

where P_y_ is the dissipated energy and C_y_ is an information transmitted. Shannon’s channel capacity equation describes the transmitted information (C_y_) connected with the bandwidth of the channel (d_space_), the dissipated energy (P_y_), and the thermal noise (N_y_):

(2) $$C_{y}=d_{space}log_{2}\left(\frac{P_{y}}{N_{y}}+1\right)$$

The absolute efficient molecular machines dissipate a minimal quantity of energy for a bit of information that is determined with the following equation:

(3) $$\varepsilon_{min}=k_{B}Tln2$$

where k_B_ is Boltzmann constant and T is temperature. Thus, the efficiency of the molecular machine is as follows:

(4) $$\epsilon_{t}=\frac{\varepsilon_{min}}{\varepsilon }$$

In relation to DNA-protein complexes, the maximal efficiencies of protein binding were calculated to be no more than 70% [48,49]. We applied this theoretical background to our results. Equation (2) was transformed to:

(5) $$C_{y}^{\prime }=L_{SC}N_{AA}log_{2}\left(\frac{N_{PC}E_{PC}}{RT}+1\right)$$

where L_SC_ and N_AA_ are mean length of sidechain and number of amino acids in 4 Å proximity to polar contacts, correspondingly; L_SC_N_AA_ is an analogue of the bandwidth of the channel from Equation (2). N_PC_ is the number of polar contacts; E_PC_ is energy of one polar contact; N_PC_∙E_PC_ represents a rough estimation of the energy that is to be dissipated (P_y_). RT is a rough estimation of thermal noise (N_y_), with R a gas constant and T as temperature. We used the following parameters: E_PC_ = 6 kJ/mol (1/2 from “ideal” H-bond in protein) and T = 298 K. The parameter C_y_′ reflects energy transfer by the protein part of the interface; this parameter was calculated for aptamer–protein and DNA helix–protein complexes. C_y_’ values are listed in Tables S3 and S5.

A question remains of how parameter C_y_′ is connected with −ΔG_b_. Changes in Gibbs free energy during binding can be represented as a sum of energy of polar contacts (P_y_ = N_PC_E_PC_) and a summand W, which includes energy of dehydration, conformational rearrangement, and energy from other types of interactions:

(6) $$-\Delta G_{b}=N_{PC}E_{PC}+W$$

From Equations (1) and (6), it follows, that:

(7) $$C_{y}^{\prime }=\frac{P_{y}}{\varepsilon }=\frac{-\Delta G_{b}}{\varepsilon }-\frac{W}{\varepsilon }$$

Supposing some of the complexes to be the most efficient (ϵ_t_ = 70% according to [49]), for those complexes, the value ε can be replaced with a constant value according to Equation (4):

(8) $$C_{y}^{\prime }=k\frac{0.7}{RTln2}\left(-\Delta G_{b}^{max}\right)-k\frac{0.7}{RTln2}W$$

**Figure 5:**
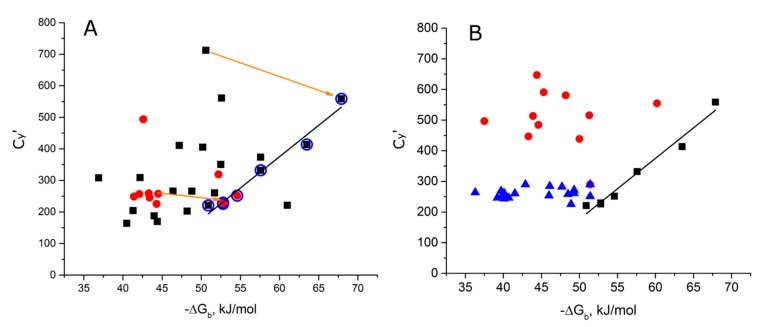
Capacity of energy transfer (C_y_′) plotted versus changes in Gibbs free energy. (**A**) Aptamer–protein complexes: G-quadruplex aptamers to thrombin are shown as red dots; all other aptamers are shown as black dots; and 7 dots chosen for linearization are shown with blue circles. The linear dependence is described with the equation y = 19.9x − 818 with R^2^ = 0.97. Examples of improvement of aptamer affinity are shown with orange arrows that are drawn from non-optimal to optimal complexes. (**B**) DNA helix–protein complexes: HTH-type proteins are shown as red dots; complexes of fis protein with different DNA helices are shown as blue dots; and linearized efficient aptamer–protein complexes are shown in black dots.

Here, Boltzmann constant was replaced with gas constant as Gibbs energy values are used as kJ per mole; k is coefficient of proportionality. From Equation (8), it follows that changes in Gibbs free energy are in linear dependence from the capacity of energy dissipation (C_y_′), if the summand W is equal for different complexes. The summand W includes all changes in energetic state of the molecule other than polar contacts, and this summand varies significantly. The example with different complexes of fis protein (Figure 5B) clearly shows that all non-optimal complexes locate at the left side from the line for optimal complexes, reflecting the high impact of energy consuming processes. Using this observation, we chose aptamer–protein complexes that located at the right side of the diagonal distribution (Figure 5A). Seven dots can be approximated with a straight line (R^2^ = 0.97) for the most efficient complexes, and all other dots are located left of the line with the single outlier. The outlier is the complex of aptamer SL5 with its protein target (the dot is in right bottom part of the Figure 5A) that is a perfect example of energy dissipation by nucleic acid component, which is discussed further in the text.

Using the representation C_y_′ versus −ΔG_b_ it is easier to compare different types of complexes, as in this case data for G-quadruplex aptamers with thrombin, other aptamer–protein complexes and HTH-type proteins with DNA duplexes are in the same range of values. Here the most efficient complexes are assumed to dissipate the energy from polar contacts without energy-consuming conformational changes. In the examples with high affinity, nucleic acids provide a complimentary surface (large numbers of polar contacts) with an appropriate protein site (with large amino acids in the interface). 

The efficiency of the sub-optimal complex can be enhanced via modification of the aptamer. An excellent example is optimization of aptamer AF113-1 into AF113-18 that led to 15 kJ/mole increase in −ΔG_b_ value (see the upper arrow in Figure 5A). Also, a clear example of efficiency improvement of the complex can be illustrated for aptamer HD1, which is to break 2 hydrogen bonds in a thymine-pair during complex formation (roughly 12 kJ/mole); the dot for its complex is located 10.5 kJ/mole left from the linear dependence (see the bottom arrow in Figure 5A). The −ΔG_b_ value was improved through manipulation of the aptamer structure only; the protein part of the interface was the same. The most efficient complexes have an additional duplex module tightly stacked to the G-quadruplex (RE31 and NU172) or just a long substituent in thymine from the thymine pair (T4K) that has no contact with a protein but is exposed to the solvent (Figure S1). Impairment of the stacking between duplex and G-quadruplex modules or replacement of the long substituent with an aromatic anchor led to the decrease in −ΔG_b_ value, respectively [15]. These tiny effects revealed that the nucleic acid component plays a significant role in energy dissipation, along with the protein component. 

One more excellent example is the single outlier with the highest efficiency of the complex, aptamer SL5. The −ΔG_b_ value for SL5 is 10 kJ/mole greater than for its counterpart SL4. The only difference between these two modified aptamers is the residue in 5′-position of dU8: isobutyl (4 carbon atoms) in SL4 and benzyl (7 carbon atoms) in SL5. The protein parts of the interfaces are the same, and aptamer conformations and thermal stability are the same [50]. The only difference is in the residues within 4Å vicinity of the “hot spot” residue, dU17. In the case of SL4, dU8 is not in contact with dU17; but in the case of SL5, benzyl substituent of dU8 does have contact with the “hot spot” residue (Figure S2). This example clearly indicates robustness of hydrophobic modifications of nucleic acids for affinity improvement with a possible role in energy transfer.

Besides graphical representation, numerical estimation of the efficiency of the complex can be used. Referring to 70% as the limit of efficiency of DNA binding proteins [48,49], we assume the parameter ϵ_t_ to be 0.7 for those 7 complexes that are located on the right edge of the C_y_′ vs. −ΔG_b_ distribution. Using parameter C_y_′ (Equation 5) and its dependence on −ΔG_b_ (Equation 8), the theoretically achievable changes in Gibbs free energy can be calculated for all DNA-protein and aptamer–protein complexes:

(9) $$-\Delta G_{b}^{lim}=\frac{1}{a}L_{SC}N_{AA}log_{2}\left(\frac{N_{PC}E_{PC}}{RT}+1\right)+\frac{b}{a}$$

where a and b are parameters from linearization of the most efficient complexes from Figure 5 (a = 19.9, and b = 818; R^2^ = 0.97). The efficiency of each complex was calculated by dividing experimental values of changes in Gibbs free energy ($\Delta G_{b}^{exp}$) by the theoretically achievable value ($\Delta G_{b}^{lim}$):

(10) $$\epsilon_{t}=\frac{\Delta G_{b}^{exp}}{\Delta G_{b}^{lim}}0.7$$

where the coefficient 0.7 reflects the 70% limit of efficiency. The data are shown in Tables S3 and S5. It is clear from both numerical analysis (Tables S3 and S5) and graphical representation (Figure 5) that many aptamer complexes and almost all natural complexes of HTH-type protein complexes are suboptimal and obviously can be improved to achieve $\Delta G_{b}^{lim}$ values. Moreover, these values can be exceeded if the aptamer “hot spots” are modified to enhance energy dissipation, as for SL5 or thrombin aptamers. 

## 4. Materials and Methods

The structures were uploaded from RCSB PDB [1] and processed with Pymol software (v.1.74) (Schrödinger, Cambridge, MA, USA) [13]. The details of amino acid selection are provided in appropriate sections. Lengths of sidechain were calculated as numbers of atoms in Cα substituents of amino acids, excluding hydrogen atoms (i.e., number of C, N, O, and S). Numbers of carbon atoms were calculated as numbers of carbon atoms in Cα substituents of aromatic or aliphatic amino acids. Hydrogen atoms were not counted. The data treatment and figure construction were made in Origin 2015 (OriginLab, Northampton, MA, USA).

## 5. Conclusions

Detailed analysis and description of 63 protein structures discriminated peculiarities of high-affinity nucleic acid–protein complexes. The volume of the amino acid sidechain within the interface was demonstrated to be the most significant parameter that correlates with affinity of aptamers to proteins. This correlation could be explained in terms of need of efficient energy transfer. A parameter for estimation of the efficiency for nucleic acid–protein complexes was proposed. These results are of great interest both for understanding the fundamental principles of protein functioning and for design and improvement of efficient ligands, particularly nucleic acid aptamers.

## Figures and Tables

**Table 1:** A list of aptamer–protein complexes and their parameters, including a host organism of protein, accession number in Protein data bank (PDB Id), resolution of the structure, numbers of nucleotides (#N) and amino acid (#AA) residues, change in Gibbs free energy during binding (ΔG_b_), kinetic constants of association (k_a_), and dissociation (k_d_). G-quadruplex aptamers to thrombin are shown in grey color. The values in brackets are references for kinetic constants.

Aptamer	Protein	Organism	PDB Id	Resolu-tion, Å	#N	#AA	−ΔG_b_, kJ/mol [16]	k_a_, M^−1^ s^−1^	k_d_, s^−1^
RNA-2	30S ribosomal protein S8	Bacillus anthracis	4PDB	2.6	38	155	36.9	-	-
NOX-D20	C5a complement anaphylatoxin	Mus musculus	4WB2	1.8	40	79	63.5	5 × 10^6^	1.0 × 10^−4^ [17]
RB011	Ectonucleotide pyrophosphatase/phosphodiesterase family member 2	Mus musculus	5HRT	2.0	34	831	50.2	-	-
NOXE36	C-C motif chemokine 2	Homo sapiens	4R8I	2.05	40	77	52.5	2 × 10^6^	2.7 × 10^−3^ [18]
Anti-Fc	Ig gamma-1 chain C region	Homo sapiens	3AGV	2.15	24	211	40.5	3 × 10^4^	3.3 × 10^−3^ [19]
SL1025	Interleukin-6	Homo sapiens	4NI7	2.4	32	186	57.6	1.2 × 10^5^	2.8 × 10^−5^ [20]
SL1025	Interleukin-6	Homo sapiens	4NI9	2.55	32	186	57.6	1.2 × 10^5^	2.8 × 10^−5^ [20]
SL1067	Interleukin-1 alpha	Homo sapiens	5UC6	2.1	23	159	46.4	-	-
2008s	L-lactate dehydrogenase	Plasmodium falciparum	3ZH2	2.1	35	316	42.2	2.8 × 10^6^	1.6 × 10^−1^ [21]
pL1	L-lactate dehydrogenase	Plasmodium vivax	5HTO	1.9	34	346	44.4	-	-
pL1	L-lactate dehydrogenase	Plasmodium vivax	5HRU	1.71	32	346	44.4	-	-
MinF	Lysozyme C	Gallus gallus	4M6D	2.68	45	129	41.3	-	-
MinE	Lysozyme C	Gallus gallus	4M4O	2	49	129	44.0	-	-
SL1049	Beta-nerve growth factor	Homo sapiens	4ZBN	2.45	28	120	57.6	8 × 10^5^	2.5 × 10^−4^ [22]
αp50RNA	Nuclear factor NF-kappa-B p105 subunit	Mus musculus	1OOA	2.45	29	326	47.2	-	-
SL4	Platelet-derived growth factor subunit B	Homo sapiens	4HQX	2.3	24	102	50.9	-	-
SL5	Platelet-derived growth factor subunit B	Homo sapiens	4HQU	2.2	24	109	61.0	-	-
ARC1172	von Willebrand Factor	Homo sapiens	3HXQ	2.69	42	209	52.6	-	-
ARC1172	von Willebrand Factor	Homo sapiens	3HXO	2.4	42	209	52.6	-	-
F5	MS2 protein capsid	Escherichia phage MS2	5MSF	2.8	18	129	48.8	-	-
F6	MS2 protein capsid	Escherichia phage MS2	6MSF	2.8	14	129	48.2	-	-
F5/2AP10	MS2 protein capsid	Escherichia phage MS2	1U1Y	2.85	17	129	51.7	-	-
HD1 (K^+^)	Thrombin	Homo sapiens	4DII	2.05	15	295	44.5	2.0 × 10^5^	3.4 × 10^−3^ [23]
T4W	Thrombin	Homo sapiens	6EO6	1.69	15	295	52.2	-	-
T4K	Thrombin	Homo sapiens	6EO7	2.24	15	295	54.6	-	-
mTBA	Thrombin	Homo sapiens	3QLP	2.14	15	295	43.4	-	-
RE31	Thrombin	Homo sapiens	5CMX	2.98	31	295	52.8	1.1 × 10^7^	6.2 × 10^−3^ [23]
HD1 (Na^+^)	Thrombin	Homo sapiens	4DIH	1.8	15	295	43.3	-	-
HD1-ΔT3	Thrombin	Homo sapiens	4LZ4	2.56	15	295	41.4	4.2 × 10^6^	9.3 × 10^−2^ [24]
HD1-ΔT12	Thrombin	Homo sapiens	4LZ1	1.65	15	295	42.1	4.2 × 10^8^	2.2 × 10^1^ [24]
NU172 (Na^+^)	Thrombin	Homo sapiens	6GN7	2.8	26	295	44.3	-	-
NU172 (K^+^)	Thrombin	Homo sapiens	6EVV	2.5	26	295	52.8	8.1 × 10^6^	3.1 × 10^−3^ [25]
AF113-1	Thrombin	Homo sapiens	3DD2	1.9	26	295	50.6	2.9 × 10^5^	5.3 × 10^−4^ [26]
AF113-18	Thrombin	Homo sapiens	5DO4	1.86	25	295	67.9	7.3 × 10^5^	1.3 × 10^−6^ [26]
HD22	Thrombin	Homo sapiens	4I7Y	2.4	27	295	42.6	4.4 × 10^5^	1.5 × 10^−3^ [27]

**Table 2:** Aptamer–protein complexes. The values of coefficient of determination, R^2^, for the linear regression curves for dependencies of different interface parameters versus changes in Gibbs free energy during binding. The strongest correlation is highlighted.

Parameters of AA within 4 Å Vicinity of Polar Contacts	All Aptamers	Excluding GQ Aptamers to Thrombin	GQ APTAMERS to thrombin
Number of polar contacts	0.02	0.04	0.04
Number of AA	0.09	0.08	0.05
Mean length of sidechain	0.05	0.49	0.01
Total atoms in AA	0.18	0.26	0.04
% of HP AA	0.03	0.03	0.09
Number of AA in PC	0.03	0.02	0.02
Mean length of SC of AA in PC	0.01	0.23	0.04
Total atoms in AA in PC	0.07	0.17	0.04
Number of AA in HS vicinity	0.01	0.02	0.06
Mean length of SC of AA in HS	0.11	0.47	0.13
Total atoms in AA in HS vicinity	0.09	0.34	0.07
Number of aromatic AA	0.05	0.14	0.03
Number of positively charged AA	0.14	0.17	0.02

AA, amino acid residues; HP AA, hydrophobic amino acid residues. PC, polar contacts; SC, sidechain; HS, ′hot spot′; GQ, G-quadruplex.

**Table 3:** Analysis of kinetic parameters of aptamer–protein complexes. The values of coefficient of determination, R^2^, for the logarithmic regression curves for the dependencies of different structural parameters versus kinetic constants of association (k_on_) and dissociation (k_off_). The strongest correlations are highlighted.

Parameters of AA within 4 Å Vicinity of Polar Contacts	k_on_	k_off_
Number of polar contacts	0.63	0.02
Number of AA	0.02	0.21
Mean length of sidechain	0.05	0.27
Total atoms in AA	0.02	0.25
% of HP AA	0.07	0.14
Number of AA in PC	0.27	0.01
Mean length of SC of AA in PC	0.17	0.68
Total atoms in AA in PC	0.02	0.33
Number of AA in vicinity to HS	0.00	0.18
Mean length of SC of AA in vicinity to HS	0.02	0.62
Total atoms in AA in vicinity to HS	0.00	0.50
Number of aromatic AA	0.03	0.05
Number of positively charged AA	0.07	0.40

AA, amino acid residue; HP AA, hydrophobic amino acid residue; SC, sidechain.

**Table 4:** A list of complexes of HTH-type proteins with DNA duplexes and their parameters, including protein name, host organism of protein, DNA sequence, accession number in Protein data bank (PDB Id), resolution of the structure, numbers of nucleotides (#N) and amino acid (#AA) residues, an apparent dissociation constant (K_d_), a change in Gibbs free energy during binding (ΔG_b_), kinetic constants of association (k_a_), and dissociation (k_d_), if known.

Protein	Organism	DNA (1 strand)	PDB Id	Resolu-tion, Å	#N	#AA	K_d_, nM	−ΔG_b_, kJ/mol	k_a_, M^−1^ s^−1^	k_d_, s^−1^
Antitoxin HipB	Escherichia coli	ttatccgctctacgggataa	4Z58	2.5	20 × 2	71	0.6 [28]	50.0	-	-
Transcriptional regulator TnrA	Bacillus megaterium	cgtgtaaggaattctgacacg	4R24	2.25	21 × 2	85	11.6 [29]	45.3	-	-
Transcriptional regulator CueR	Escherichia coli	gaccttccccttgctggaaggtc	4WLW	2.8	23 × 2	135	15 [30]	44.6	-	-
DNA-binding protein fis	Escherichia coli	aaatttgtttgaattttgagcaaattt	3IV5	2.9	27 × 2	98	0.2 [31]	51.4	-	-
		aaatttgtttaaattttgagcaaattt	3JR9	2.9			0.2 [31]	51.4	-	-
		aaatttggtcatttcttaactaaattt	3JRA	3.11			8 [31]	42.9	-	-
		aaatttgtttgttttttgagcaaattt	3JRB	3.1			0.5 [31]	49.3	-	-
		aaatttgtttgggcgctgagcaaattt	3JRC	3.08			140 [31]	36.3	-	-
		aaatttgtttgttaaatgagcaaattt	3JRD	3.1			1 [31]	47.7	-	-
		aaatttgtttgaaaaatgagcaaattt	3JRE	3.17			0.5 [31]	49.3	-	-
		aaatttgtttgaactttgagcaaattt	3JRF	3.05			0.6 [31]	48.9	-	-
		aaatttgttggaattttcagcaaattt	3JRG	3.11			2 [31]	46.1	-	-
		aaatttgtttcaatttggagcaaattt	3JRH	2.88			40 [31]	39.2	-	-
		aaatttgttgtaatttgtagcaaattt	3JRI	3.11			33 [31]	39.7	-	-
		aaatttggaggaattttctccaaattt	5E3O	2.78			28 [32]	40.1	-	-
		aaatttgtaggaattttctgcaaattt	5E3N	2.66			21 [32]	40.7	-	-
		aaattagtttgaatctcgagctaattt	5E3M	2.89			15 [32]	41.5	-	-
		aaattggtttgaattttgagccaattt	5E3L	2.66			30 [32]	39.9	-	-
		aaattcgtttgaattttgagcgaattt	5DTD	2.64			2.1 [32]	46.0	-	-
		aaattagtttgaattttgagctaattt	5DS9	2.56			0.7 [32]	48.5	-	-
		aaatttgtttgagcgttgagcaaattt	4IHV	2.72			28 [33]	40.1	-	-
MerR family regulator protein	Hemophilus influenzae	cttagagttcactctaag	5D8C	2.25	18 × 2	137	25.2 [34]	43.3	-	-
Transcriptional regulator SinR	Bacillus subtilis	aaagttctctttagagaacaa	3ZKC	3.0	21 × 2	111	270 [35]	37.5	1 × 10^5^	2 × 10^−2^
Transcriptional regulator ygiT	Escherichia coli	agttataacctaaaaggttaattaca	3O9X	2.10	26 × 2	133	0.8 [36]	48.2	-	-
Multiple antibiotic resistance protein MarR	Escherichia coli	catacttgcctgggcaatatt	5H3R	2.67	21 × 2	147	1.0 [37]	51.3	-	-
Transcriptional repressor YvoA	Bacillus subtilis	cagtggtctagaccactgg	4WWC	2.90	19 × 2	246	0.028 [38]	60.2	1.4 × 10^7^	2.3 × 10^−4^
Heat-shock regulator CtsR	Geobacillus stearothermophilus	gattaaggtcaaatatagtcaaaata	3H0D	2.4	26 × 2	155	22 [39]	44.4	-	-
Transcriptional regulator IscR	Escherichia coli	ataaatccacacagtttgtattgttttgt	4HF1	2.22	29 × 2	170	17-24 [40]	43.9	-	-

## Supplementary Materials

The following are available online. Table S1: Interfaces of aptamer–proteins complexes. Table S2: Parameters for the set of aptamer–protein complexes that were used to find correlations. Table S3: Parameters for the set of aptamer–protein complexes that were used to find correlations (continued). Table S4: Interfaces of HTH-type proteins complexed with DNA duplexes. Table S5: Parameters for HTH-type proteins complexed with DNA duplexes that were used to find correlations. Figure S1: Thrombin complexes with DNA aptamers. Figure S2: PDGFB complexes with modified DNA aptamers SL4 and SL5.
