# Supplementary material for: Energy Transfer as A Driving Force in Nucleic Acid–Protein Interactions
Source: Molecules. 2019 Apr 11;24(7):1443. doi: 10.3390/molecules24071443 (PMC6480146; doi:10.3390/molecules24071443)
Supplement: Supplementary file 1 [file molecules-24-01443-s001.pdf]

Supplementary Materials

Table S1. Aptamer-proteins complexes. A list of nucleotides forming polar contacts (N), amino acids forming polar contacts (PC), amino acids within 4 Å vicinity of atoms in polar contacts (4 Å) and amino acids in 4Å vicinity to nucleotides forming ≥3 polar contacts (HS). If more than one conformation of complex was reported, the alternative variants of interfaces are divided with dashes.

4pdb				4wb2				5hrt				4r8i				3agv			
N	PC	4Å	HS	N	PC	4Å	HS	N	PC	4Å	HS	N	PC	4Å	HS	N	PC	4Å	HS
A4	S53	P51	A112	Oa4	R684	R684	K695	Omg7	R244	R244	K316	Og5	R18	N17	S21	G4	K340	A339	K340
G6	K54	A52	K113	Ou5	E688	E688	H696	G8	R246	G245	K467	Ou6	K19	R18	Q23	Uft6	G341	K340	G341
C7	Q80	S53	A114	Og6	S697	H696	S697	Omg9	E247	R246	F469	Oc7	I20	K19	R24	G7	Q342	G341	R344
C16	A114	K54	S132	3ka7	K701	S697	K701	A10	K316	E247		Oc9	S21	I20		-----	R344	Q342	Y373
C17	S130	I55	K133	Ou21	D705	V698	D705	Omg15	R455	K248		Ou10	V22	S21		G4	Y373	R344	L398
A24	S132	E58	T146	Og25	R708	K701	R708	Omc20	L458	K316		Oc11	Q23	V22		G7	G402	Y373	G402
A26	K133	Q80	G147	Og26	N710	Y704	R721	T21	S465	Y317		Og22	R24	Q23			-----	L398	
U27	T146	K113	G148	3ka28	R718	D705	V722	T22	K467	L384		Oa26	S63	R24			K340	D401	
	G147	A114		3ka30	R721	R708	T723	T23		A454		Og27	H66	L25			Q342	G402	
	E149	D115		Oc31	T723	V709	I724	C28		R455				W59			R344	S403	
		S130		Og32	-----	N710		T29		K456				S63			Y373	F404	
		T131		Og39	K695	F711				P457				H66			G402	-----	
		S132		-----	S697	E713				L458				Q70				A339	
		K133		Oa4	K701	R718				D459								K340	
		V135		Ou5	D705	R721				S465								G341	
		T146		Og6	R708	V722				G466								Q342	
		G147		Ou21	R721	T723				K467								R344	
		G148		3ka28	T723	I724				F469								Y373	
		E149		Og29		G725												L398	
				Oc31		-----												D401	
				Og32		Y694												G402	
				Og39		K695												S403	
						H696													
						S697													
						V698													
						K701													
						D705													
						R708													
						R721													

						V722<br>T723<br>I724													
4ni7				4ni9				5uc6				3zh2				5hto			
N	PC	4Å	HS	N	PC	4Å	HS	N	PC	4Å	HS	N	PC	4Å	HS	N	PC	4Å	HS
G5<br>Omg6<br>Duz7<br>2JU12<br>A13<br>Duz14<br>G29<br>Duz30	R16<br>R24<br>K27<br>Q28<br>R30<br>S118<br>K128<br>K171<br>Q175	R16<br>L19<br>R24<br>D26<br>K27<br>Q28<br>R30<br>Y31<br>A114<br>S118<br>V121<br>K128<br>K171<br>Q175	K27<br>Q28<br>R30<br>Y31<br>D34<br>M117<br>S118<br>V121	2JU12<br>A13<br>Duz14<br>G29<br>-----<br>G5<br>Omg6<br>Duz7<br>2JU12<br>A13<br>Duz14	R16<br>K27<br>Q28<br>R30<br>S118<br>-----<br>R24<br>K27<br>Q28<br>R30<br>Y31<br>S118<br>Q124<br>K128	R16<br>L19<br>R24<br>D26<br>K27<br>Q28<br>R30<br>Y31<br>A114<br>S118<br>V121<br>F125<br>K128<br>-----<br>R24<br>D26<br>K27<br>Q28<br>R30<br>Y31<br>A114<br>M117<br>S118<br>V121<br>Q124<br>F125<br>K128	K27<br>Q28<br>R30<br>Y31<br>D34<br>M117<br>S118<br>V121	85y7<br>G11<br>85y14	R16<br>S61<br>D64<br>D65<br>K67<br>I68<br>W113	R16<br>K60<br>S61<br>S62<br>K63<br>D64<br>D65<br>A66<br>K67<br>I68<br>T69<br>W113<br>E114<br>T115	K60<br>S61<br>K63<br>D64<br>D65<br>A66<br>K67<br>I68<br>W113<br>T115	C6<br>G7<br>G8<br>T9<br>A10<br>G11<br>G19<br>A22<br>-----<br>C6<br>G7<br>G8<br>T9<br>A10<br>G11<br>G19<br>A22<br>-----<br>G4<br>C6<br>G7<br>G8<br>T9<br>A10<br>G11<br>A18<br>A22	D35<br>I36<br>K84<br>A85<br>G87<br>K88<br>S89<br>D90<br>H232<br>-----<br>D35<br>I36<br>K84<br>A85<br>G87<br>K88<br>S89<br>D90<br>H232<br>-----<br>D35<br>I36<br>K84<br>A85<br>G87<br>K88<br>S89<br>D90<br>K91<br>H232	F34<br>D35<br>I36<br>V37<br>A80<br>F82<br>K84<br>A85<br>P86<br>G87<br>K88<br>S89<br>D90<br>H232<br>-----<br>F34<br>D35<br>I36<br>V37<br>A80<br>F82<br>K84<br>A85<br>P86<br>G87<br>K88<br>S89<br>D90<br>H232<br>-----<br>F34<br>D35<br>I36<br>V37<br>A80	K84<br>A85<br>P86<br>G87<br>K88<br>S89<br>D90	G10<br>T12<br>G23<br>-----<br>T8<br>G10<br>T12<br>G23	K44<br>T83<br>-----<br>K44<br>G81<br>T83	S12<br>G13<br>M14<br>K44<br>F82<br>T83<br>K84<br>L98<br>-----<br>S12<br>G13<br>M14<br>K44<br>T79<br>A80<br>G81<br>F82<br>T83<br>K84<br>L98	-----

														K84<br>A85<br>P86<br>G87<br>K88<br>S89<br>D90<br>K91<br>W93<br>H232					
5hru				4m6d				4m4o				4zbn				1ooa			
N	PC	4Å	HS	N	PC	4Å	HS	N	PC	4Å	HS	N	PC	4Å	HS	N	PC	4Å	HS
T8<br>G10<br>A11<br>T12<br>G23	D35<br>K44<br>G81<br>T83<br>Y238	G11<br>S12<br>G13<br>M14<br>F34<br>D35<br>K44<br>T79<br>A80<br>G81<br>F82<br>T83<br>K84<br>L98<br>Y238	-----	U30<br>A31<br>A35<br>G36<br>C39<br>-----<br>U30<br>A31<br>A35<br>G36<br>-----<br>U30<br>A31<br>A35<br>G36	K1<br>C6<br>E7<br>R14<br>R128<br>-----<br>K1<br>R5<br>C6<br>E7<br>R128<br>-----<br>K1<br>R5<br>E7<br>R128	K1<br>R5<br>C6<br>E7<br>L8<br>A9<br>A10<br>R14<br>G126<br>C127<br>R128<br>L129<br>-----<br>K1<br>R5<br>C6<br>E7<br>L8<br>A9<br>A10<br>R125<br>C127<br>R128<br>L129<br>-----<br>K1	R5<br>C6<br>E7<br>A10<br>G126<br>C127<br>R128	U30<br>A31<br>A35<br>G36	K1<br>R5<br>E7<br>R128	K1<br>R5<br>C6<br>E7<br>A10<br>R125<br>G126<br>C127<br>R128<br>L129	R5<br>C6<br>E7<br>A10<br>G126<br>C127<br>R128	Duz10<br>Duz11<br>Duz18<br>C21<br>C22<br>G23<br>Duz24<br>Duz27<br>-----<br>Duz10<br>Duz11<br>Duz18<br>C21<br>C22<br>G23<br>Duz24<br>Duz27	S17<br>S19<br>W21<br>K32<br>R59<br>R69<br>R103<br>-----<br>S17<br>S19<br>W21<br>K32<br>R59<br>R69<br>R103	F12<br>D16<br>S17<br>V18<br>S19<br>V20<br>W21<br>V22<br>D30<br>Ile31<br>K32<br>G33<br>F54<br>R59<br>R69<br>H84<br>R103<br>-----<br>F12<br>D16<br>S17<br>V18<br>S19<br>V20<br>W21<br>V22	-----	G8<br>A9<br>A11<br>C12<br>G14<br>A17<br>G19<br>U20<br>U21<br>G22<br>G23<br>C24<br>G25<br>-----<br>G8<br>A9<br>A11<br>C12<br>U15<br>A17<br>G19<br>U20<br>U21<br>G22<br>G23<br>C24	R54<br>R56<br>Y57<br>E60<br>P62<br>S63<br>H64<br>K77<br>K144<br>K145<br>K241<br>K249<br>R305<br>-----<br>R54<br>R56<br>Y57<br>E60<br>S63<br>K77<br>K144<br>K145<br>K241	R54<br>R56<br>Y57<br>E60<br>G61<br>P62<br>S63<br>H64<br>G65<br>K77<br>H141<br>V142<br>T143<br>K144<br>K145<br>K241<br>K249<br>R305<br>-----<br>R54<br>R56<br>Y57<br>E60<br>G61<br>P62<br>S63	Y57<br>H141<br>T143<br>K144

						R5<br>C6<br>E7<br>A10<br>R125<br>C127<br>R128<br>L129								D30<br>Ile31<br>K32<br>G33<br>F54<br>R59<br>R69<br>H84<br>R103		G25		H64<br>G65<br>K77<br>H141<br>T143<br>K144<br>K145<br>K241	
4hqx				4hqu				3dd2				5do4				4i7y			
N	PC	4Å	HS	N	PC	4Å	HS	N	PC	4Å	HS	N	PC	4Å	HS	N	PC	4Å	HS
Duz1<br>Ubi8<br>Upe17<br>A19	V39<br>R73<br>K80<br>F84	L38<br>V39<br>W40<br>R73<br>K80<br>K81<br>P82<br>I83<br>F84<br>K85	L38<br>V39<br>W40<br>R73<br>I75<br>F84	Duz1<br>Duz8<br>Upe17<br>A19	V39<br>R73<br>K80<br>F84	L38<br>V39<br>W40<br>R73<br>K80<br>K81<br>P82<br>I83<br>F84<br>K85	L38<br>V39<br>W40<br>R73<br>I75<br>F84	Cfl6<br>A7<br>A8<br>A9<br>Cfl11<br>G13<br>A15<br>G16<br>Uft17	H91<br>R101<br>R126<br>Q131<br>R165<br>K169<br>D178<br>N179<br>R233<br>K236<br>K240	H91<br>P92<br>R93<br>D100<br>R101<br>R126<br>E127<br>A129<br>L130<br>Q131<br>V163<br>E164<br>R165<br>P166<br>K169<br>D178<br>N179<br>M180<br>H230<br>R233<br>L234<br>K235<br>K236<br>W237<br>I238<br>K240	H91<br>R93<br>R101<br>N179<br>R233<br>L234<br>K236<br>W237	A7<br>A8<br>Cfz11<br>G13<br>A14<br>A15<br>G16	D34<br>R36<br>R98<br>Q131<br>R170<br>K174<br>D183<br>N184<br>R245<br>W249	D34<br>G35<br>R36<br>D97<br>R98<br>A127<br>L130<br>Q131<br>A132<br>V168<br>E169<br>R170<br>P171<br>K174<br>D183<br>N184<br>M185<br>H242<br>R245<br>L246<br>K247<br>K248<br>W249<br>I250	H87<br>R89<br>R98<br>N184<br>R245<br>L246<br>K248<br>W249	T9<br>T18<br>G20<br>G21<br>G23<br>T24<br>G25<br>C27	P92<br>R93<br>N95<br>R97<br>R101<br>R126<br>K169<br>H230<br>R233<br>W237	I90<br>H91<br>P92<br>R93<br>Y94<br>N95<br>R97<br>E97A<br>N98<br>R101<br>R126<br>K169<br>H230<br>R233<br>W237<br>V241	P92<br>R93<br>Y94<br>N95<br>W96<br>R97<br>R101
6eo6				6eo7				3qlp				5cmx				4dii			
N	PC	4Å	HS	N	PC	4Å	HS	N	PC	4Å	HS	N	PC	4Å	HS	N	PC	4Å	HS

77y4<br>G5<br>T12<br>T13<br>G14	T432<br>R433<br>Y434<br>E435<br>R436<br>N437<br>Y477	R425<br>H429<br>S430<br>T432<br>R433<br>Y434<br>E435<br>R436<br>N437<br>I438<br>Y477	I371<br>H429<br>R433<br>E435<br>R436<br>N437<br>I438<br>Y477	8dt4<br>T12<br>T13<br>G14	R433<br>Y434<br>E435<br>R436<br>N437<br>Y477	H429<br>S430<br>T432<br>R433<br>Y434<br>E435<br>R436<br>N437<br>Y477	I371<br>H429<br>R433<br>E435<br>R436<br>N437<br>I438<br>Y477	G2<br>T4<br>T12<br>T13<br>G14	R75<br>Y76<br>E77<br>R77A<br>N78<br>Y117	H71<br>S72<br>T74<br>R75<br>Y76<br>E77<br>R77A<br>N78<br>Y117	I24<br>T74<br>R75<br>Y76<br>E77<br>R77A<br>N78<br>I79<br>Y117	T11<br>T12<br>G13<br>T21	R75<br>Y76<br>E77<br>R77A<br>Y117	H71<br>S72<br>T74<br>R75<br>Y76<br>E77<br>R77A<br>N78<br>Y117	R75<br>Y76<br>R77A	T3<br>T4<br>G5<br>T13	H71<br>R75<br>Y76<br>R77A<br>N78<br>Y117	I24<br>H71<br>T74<br>R75<br>Y76<br>E77<br>R77A<br>N78<br>I79<br>Y117	T74<br>R75<br>Y76<br>R77A<br>N78<br>I79
4dih				4lz4				4lz1				3hxq				3hxo			
N	PC	4Å	HS	N	PC	4Å	HS	N	PC	4Å	HS	N	PC	4Å	HS	N	PC	4Å	HS
T3<br>T4<br>G5<br>T13<br>G14	R75<br>Y76<br>E77<br>R77A<br>Y117	H71<br>S72<br>T74<br>R75<br>Y76<br>E77<br>R77A<br>N78<br>I79<br>Y117	I24<br>R75<br>E77<br>R77A<br>N78<br>I79<br>Y117	T4<br>T12<br>T13<br>G14<br>-----<br>T4<br>T12<br>T13<br>G14	R75<br>Y76<br>E77<br>R77A<br>N78<br>Y117<br>-----<br>R75<br>Y76<br>E77<br>R77A<br>N78<br>Y117	H71<br>S72<br>T74<br>R75<br>Y76<br>E77<br>R77A<br>N78<br>Y117<br>-----<br>H71<br>S72<br>T74<br>R75<br>Y76<br>E77<br>R77A<br>N78<br>Y117	I24<br>R75<br>E77<br>R77A<br>N78<br>I79<br>Y117	T3<br>T4<br>G5<br>T13<br>G14	R75<br>Y76<br>E77<br>R77A<br>N78<br>Y117	H71<br>S72<br>T74<br>R75<br>Y76<br>E77<br>R77A<br>N78<br>Y117	I24<br>R75<br>E77<br>R77A<br>N78<br>I79<br>Y117	C7<br>A8<br>G9<br>T10<br>G11<br>G21<br>T22<br>C24<br>G25<br>G26<br>T27<br>G28<br>C29<br>C30<br>C32	R524<br>Q625<br>E626<br>Q628<br>R629<br>R632<br>N633<br>R636<br>Y637<br>P655<br>A657<br>L659<br>K660	R524<br>K599<br>F603<br>Q625<br>E626<br>P627<br>Q628<br>R629<br>M630<br>S631<br>R632<br>N633<br>R636<br>Y637<br>P655<br>H656<br>A657<br>N658<br>L659<br>K660<br>Q661	Q625<br>Q628<br>R629<br>S631<br>R632<br>F655<br>H656<br>A657<br>N658<br>L659<br>K660	C7<br>A8<br>T10<br>G21<br>T22<br>C24<br>G25<br>G26<br>T27<br>G28<br>C29<br>T31<br>C32	E626<br>Q628<br>R629<br>R632<br>N633<br>Y637<br>P655<br>A657<br>L659<br>K660	K599<br>Q625<br>E626<br>P627<br>Q628<br>R629<br>M630<br>S631<br>R632<br>N633<br>Y637<br>P655<br>H656<br>A657<br>N658<br>L659<br>K660<br>Q661<br>L664	Q625<br>Q628<br>R629<br>S631<br>R632<br>F655<br>H656<br>A657<br>N658<br>L659<br>K660
5msf				1u1y				6msf				6gn7				6evv			
N	PC	4Å	HS	N	PC	4Å	HS	N	PC	4Å	HS	N	PC	4Å	HS	N	PC	4Å	HS
C2<br>C10<br>A11	K43<br>T45<br>S47	K43<br>T45<br>C46	V29<br>K43<br>T45	C2<br>C10<br>A11	K43<br>T45<br>S47	K43<br>T45<br>C46	V29<br>K43<br>T45	C8<br>A9<br>-----	T45<br>S47<br>T59	T45<br>C46<br>S47	V29<br>K43<br>T45	G8<br>T10<br>G13	R75<br>Y76<br>E77	I24<br>H71<br>S72	R75<br>Y76<br>R77A	T10<br>G13<br>G18	R75<br>Y76<br>E77	H71<br>S72<br>T74	R75<br>Y76<br>R77A

-----<br>C10<br>A11	R49<br>T59<br>K61<br>E63<br>Y85<br>N87<br>-----<br>K43<br>T45<br>S47<br>T59<br>E63<br>Y85	S47<br>V48<br>R49<br>S51<br>K57<br>T59<br>I60<br>K61<br>E63<br>R83<br>Y85<br>N87<br>-----<br>K43<br>T45<br>C46<br>S47<br>V48<br>T59<br>I60<br>K61<br>E63<br>R83<br>Y85	C46<br>S47<br>T59<br>K61<br>E63<br>Y85<br>N87		R49<br>T59<br>K61<br>E63<br>Y85<br>-----<br>K43<br>T45<br>S47<br>T59<br>K61<br>E63<br>Y85	S47<br>V48<br>R49<br>S51<br>K57<br>T59<br>I60<br>K61<br>E63<br>R83<br>Y85<br>N87<br>-----<br>K43<br>T45<br>C46<br>S47<br>V48<br>T59<br>I60<br>K61<br>E63<br>R83<br>Y85	C46<br>S47<br>T59<br>K61<br>E63<br>Y85<br>N87	U7<br>C8<br>A9	E63<br>Y85<br>N85<br>-----<br>K43<br>T45<br>S47<br>T59<br>Y85	V48<br>T59<br>I60<br>K61<br>E63<br>R83<br>Y85<br>N87<br>-----<br>K43<br>T45<br>C46<br>S47<br>V48<br>T59<br>I60<br>K61<br>E63<br>R83<br>Y85	C46<br>S47<br>T59<br>K61	G18<br>T19<br>G20	R77A	T74<br>R75<br>Y76<br>E77<br>R77A<br>N78<br>I79	N78<br>I79	T19<br>G20	R77A<br>Y117	R75<br>Y76<br>E77<br>R77A<br>N78<br>Y117	

Table S2. Data from literature (ΔG_b) and calculated data (other parameters) for the set of aptamer-protein complexes that were used to find correlations: changes in Gibbs free energy during binding (ΔG_b) and characteristics of interfaces: number of polar contacts (PC), number of amino acids (AA) and hydrophobic amino acids (HP AA) within 4 Å vicinity of PC, mean length of sidechain (SC) in AA and HP AA, total atoms in AA and HP AA, proportion of HP AA, number of aliphatic and aromatic carbon atoms ('carbons') in AA and HP AA, proportion of 'carbons' in HP AA versus total AA, mean number of 'carbons' in AA and HP AA. Hydrogen atoms were not counted. Results for alternative conformations were averaged.

PDB id	$-\Delta G_b$, kJ/mol	Number of polar contacts	Number of AA	Mean length of sidechain	Number of HP AA	Mean length of SC of HP AA	% of HP AA	Total atoms in AA	Total atoms in HP AA	Carbons in AA	Carbons in HP AA	% of carbons in HP AA	Mean carbons in AA	Mean carbons in HP AA
4pdb	36.9	16	19	3.05	5	2.40	26.3	58	12	38	12	31.6	2.00	2.40
4wb2	63.5	20	15.5	4.74	5	4.58	32.5	73.5	23	47.5	22	45.9	3.10	4.38
5hrt	50.2	14	18	4.39	6	4.50	33.3	79	27	55	26	47.3	3.06	4.33
4r8i	52.5	18	13	4.92	4	5.25	30.8	64	21	41	20	48.8	3.15	5.00
3agv	40.5	7	10.5	3.75	3.5	4.67	33.2	39.5	16.5	26.5	16	59.4	2.51	4.50
4ni7-4ni9	57.6	13.7	13.3	4.90	4.7	4.40	35.2	65.3	20.7	42	19.3	46.0	3.15	4.12
5uc6	46.4	9	14	4.21	3	5.00	21.4	59	15	39	14	35.9	2.79	4.67
3zh2	42.2	18.5	14.5	3.86	7	3.93	48.3	56	27.5	44	27	61.5	3.03	3.86
5hto-5hru	44.4	5.3	12.3	3.64	5.3	4.95	42.1	45	26.7	37.7	25	64.1	3.05	4.65
4m6d	41.3	10	10.7	4.10	5.3	2.31	49.7	43.3	12.3	25.3	10.3	40.2	2.38	1.92
4m4o	44.0	7	10	4.50	4	2.25	40.0	45	9	24	7	29.2	2.40	1.75
4zbn	57.6	11	16.5	4.73	7	5.29	42.4	78	37	55	36	65.5	3.33	5.14
1ooa	47.2	18.5	16.5	4.51	2.5	5.08	15.0	74.5	12.5	50	11.5	22.9	3.03	4.67
4hqx	50.9	7	10	5.30	6	5.17	60.0	53	31	45	30	66.7	4.50	5.00
4hqu	61.0	7	10	5.30	6	5.17	60.0	53	31	45	30	66.7	4.50	5.00
3hxq-3hxo	52.6	25	20	4.72	7	4.07	35.1	94.5	28.5	58.5	26.5	45.4	2.92	3.79
5msf	48.8	9.5	13.5	4.29	4	4.25	30.0	58	17	38	15.5	41.4	2.82	3.88
1u1y	51.7	10	13	4.30	4	4.25	31.5	56	17	37.5	15	40.6	2.90	3.75
6msf	48.2	7	11	4.41	4	4.25	36.4	48.5	17	32	15	47.1	2.91	3.75

3dd2	50.6	18	26	5.00	9	4.00	34.6	130	36	82	34	41.5	3.15	3.78
5do4	67.9	14	24	4.54	9	3.78	37.5	109	34	68	32	47.1	2.83	3.56
4i7y	42.6	16	16	5.81	5	5.60	31.3	93	28	57	26	45.6	3.56	5.20
6eo6	52.2	15	11	5.55	3	6.67	27.3	61	20	37	18	48.6	3.36	6.00
6eo7	54.6	13	9	5.56	2	8.00	22.2	50	16	30	14	46.7	3.33	7.00
3qlp	43.4	12	9	5.56	2	8.00	22.2	50	16	30	14	46.7	3.33	7.00
5cmx	52.8	9	9	5.56	2	8.00	22.2	50	16	30	14	46.7	3.33	7.00
4dii	44.5	11.5	9.5	5.58	4	6.00	42.1	53	24	35	22	62.9	3.68	5.50
4dih	43.3	11	10	5.40	3	6.67	30.0	54	20	34	18	52.9	3.40	6.00
4lz4	41.4	12.5	9	5.56	2	8.00	22.2	50	16	30	14	46.7	3.33	7.00
4lz1	42.1	14	9	5.56	2	8.00	22.2	50	16	30	14	46.7	3.33	7.00
6gn7	44.3	9	10	5.00	3	5.33	30.0	50	16	31	15	48.4	3.10	5.00
6evv	52.8	9.5	9	5.56	2	8.00	22.2	50	16	30	14	46.7	3.33	7.00

Table S3. Calculated data for the set of aptamer-protein complexes that were used to find correlations: characteristics of amino acids participated in polar contacts (AA in PC) and amino acids within 4 Å vicinity of hot spots (HS) that are nucleotides with ≥3 PC. The characteristics are number of amino acids (AA), mean length of sidechain (SC) in AA, total atoms in AA, sum of positively charged and aromatic amino acids (F, Y, W, H, R, K). Hydrogen atoms were not counted. Results for alternative conformations were averaged. Parameter C_y' was calculated using equation (5), mean length of SC and number of amino acids in 4Å vicinity of PC; the values used for linearization are colored. The efficiency of the complexes was calculated using equation (8); low efficient complexes (efficiency < 55%) are colored with red, highly efficient complex (efficiency >80%) is colored with green.

PDB id	Mean length of SC of AA in PC	Number of AA in PC	Total atoms in AA in PC	Mean length of SC of AA in HS	Number of AA in HS vicinity	Total atoms in HS vicinity	Sum of F, Y, W, H, R, K	C_y'	Efficiency, %
4pdb	3.00	10	30	2.13	8	17	3	308	45.6
4wb2	4.74	8.5	40.5	4.60	10	46	7	413	71.8
5hrt	5.25	8	42	5.67	3	17	9	405	57.1
4r8i	4.56	9	41	4.67	3	14	5	350	62.5
3agv	5.00	5.5	27.5	4.00	6	24	3.5	164	57.4
4ni7-4ni9	5.34	7.3	39.3	4.75	8	38	6.7	332	69.7
5uc6	5.14	7	36	4.30	10	43	5	266	59.6
3zh2	3.52	9.5	33.5	2.86	7	20	5.3	309	52.1
5hto-5hru	3.56	3.3	12	-	-	-	4.3	170	62.6
4m6d	5.47	4.7	25.3	3.43	7	24	4	204	56.2
4m4o	6.00	4	24	3.43	7	24	4	187	60.9
4zbn	5.71	7	40	-	-	-	7.5	374	67.3
1ooa	5.41	11	59.5	5.50	4	22	10	411	53.5
4hqx	5.50	4	22	5.83	6	35	6	221	68.2
4hqu	5.50	4	22	5.83	6	35	6	221	81.7
3hxq-3hxo	5.07	11.5	58.5	4.82	11	53	7.5	561	53.1
5msf	4.22	8	34	3.90	10	39	5.5	266	62.7
1u1y	4.59	7.5	34.5	3.80	10	38	5	260	66.7

6msf	3.83	6	23	3.14	7	22	3.5	202	65.8
3dd2	5.64	11	62	6.11	9	55	12	712	46.0
5do4	6.00	10	60	6.25	8	50	9	558	68.7
4i7y	6.30	10	63	6.71	7	47	10	494	45.2
6eo6	6.00	7	42	5.60	10	56	6	319	63.9
6eo7	6.50	6	39	5.63	8	45	5	251	71.1
3qlp	6.50	6	39	5.56	9	50	5	246	56.8
5cmx	7.00	5	35	7.33	3	22	5	226	70.4
4dii	6.80	5	34	5.50	6	33	4.5	257	57.6
4dih	7.00	5	35	5.57	7	39	5	259	56.0
4lz4	6.50	6	39	5.57	7	39	5	249	54.0
4lz1	6.50	6	39	6.25	8	50	5	256	54.5
6gn7	6.75	4	27	6.00	5	30	4	226	59.1
6evv	6.30	5	31.5	7.33	3	22	5	229	70.2

Table S4. HTH-type proteins complexed with DNA duplexes. A list of nucleotides forming polar contacts (N), amino acids forming polar contacts (PC), amino acids within 4 Å vicinity of atoms in polar contacts (4Å) and amino acids in 4 Å vicinity to nucleotides forming ≥3 polar contacts (HS). If more than one conformation of complex was reported, the alternative variants of interfaces are divided with dashes.

4Z58				4R24				4WLW				3IV5				5D8C			
N	PC	4Å	HS	N	PC	4Å	HS	N	PC	4Å	HS	N	PC	4Å	HS	N	PC	4Å	HS
A3	R21	R21	Q28	T12	S15	S15	I16	C14	S4	N2	R18	T5	N73	G72	G72	A11	T5	T5	T5
T2	Q28	W26	Q39	C13	G17	I16	S26	T15	T13	I3	R31	T6	Q74	N73	N73	C12	R20	A6	R20
A12'	K38	T27	T41	T14	S26	G17	R28	G16	K15	S4	G35	G7	T75	Q74	Q74	C1'	Y23	A16	R33
C13'	Q39	Q28	S43	G1'	R28	I18	R31	G17	R18	D5	R37	T8	N84	T75	T75	T2'	K24	T18	G37
G14'	T41	S29	N47	T2'	Q29	S26	Y32	G21	Y20	T13		T16'	R85	R76	R85	T3'	R33	R20	N38
G15'	S43	I37	T52	G3'	R31	E27	R43	T22	R31	S14		C17'	T87	I83	R89	A4'	R39	Y22	R39
G16'	N47	K38	T53	A10'	Y32	R28	G47	C23	N34	K15		A18'	R89	N84	-----	G5'	L62	D23	-----
	N51	Q39	T54	T11'	Y33	Q29	I48	C3'	Y36	A16		T5'	K90	R85	G72	-----	-----	K24	T5
	T53	A40			R43	R31	R49	C4'	R37	R18		T6'	-----	T87	N73	A11	T5	R33	R20
	T56	T41			T46	Y32	Q71	T5'	R54	Y20		G7'	N73	L88	Q74	C12	R20	G37	R33
		S43			R49	Y33	T72	T6'		E21		T16	Q74	R89	T75	C1'	Y23	N38	G37
		N44			T72	R36				R31		T17	T75	K90	R85	T2'	K24	R39	N38
		Q46				R43				N34		G18	N84	-----	R89	T3'	R33	R40	R39
		N47				T44				G35			R85	E59		G5'	R39	F41	
		N48				T46				Y36			T87	G72			L62	K56	

		N51				G47				R37			R89	N73				M60	
		T52				I48				T38			K90	Q74				S61	
		T53				R49				R54			K91	T75				L62	
		L54				K50				L60				R76				K63	
		T55				I66								I83				-----	
		T56				V70								N84				T4	
						Q71								R85				T5	
						T72								T87				A6	
						S73								R89				A16	
														K90				T18	
														K91				R20	
																		Y22	
																		D23	
																		K24	
																		R33	
																		G37	
																		N38	
																		R39	
																		R40	
																		F41	
																		K56	
																		M60	
																		S61	
																		L62	
																		K63	
3ZKC				3O9X				5H3R				4WWC				3H0D			
N	PC	4Å	HS	N	PC	4Å	HS	N	PC	4Å	HS	N	PC	4Å	HS	N	PC	4Å	HS
A3	R10	N41'	N41'	T14	R23	Y102'	N97	G12	Q23	S65'	Q23'	G3	I11	I9	S36	T16	N3	N3'	N3
G4	S16	R10	R10	T15	Q85	H110'	R101	G13	T39	Q23	T39	T4	Y12	P10	E37	A17	I4	R49'	I4
T5	L17	K11	S16	A16	G94	P111'		G14	Q42	T39	A41	G5	S36	I11	R38	G18	S5	N3	S5
T6	S18	Y15	L17	G17	N97	T113'		C15	V58	A40	Q42	G6	E37	Y12	R48	T5'	K27	I4	K27
A13'	A27	S16	S18	G18	S100	T21		A3'	D67	A41	V66	T7'	R38	Y13	R52	T6'	R28	S5	R28
G14'	S29	L17	K28	T19	R101	F22		A5'	R73	Q42	D67	C8'	S47	P35	R66	G8'	S29	D6	S29
A15'	Y30	S18	S29	A5'	Y102	R23		T6'	R77	T56	R73	T9'	R48	S36	G71	A12'	S40	I26	Q41
G16'	S32	E19	Y30	T6'	Q108	G24		G8'	R86	P57	M74	A10'	T50	E37	T72	T15'	Q41	K27	N43
A17'	R36	V26	S32	A7'	H110	N65		G14'	R94	V58	R77	-----	R52	R38	-----	-----	N43	R28	Y44
-----	Q39	A27	R36	A15'		R78			V96	E59		G3	R66	E39	S36	A25	Y44	S29	R49
T3	S43	K28	Q39	G16'		T84				V66		T4	G69	I46	E37	T15	R49	E30	S60
G4	-----	S29	P42			Q85				D67		G5	T72	S47	R38	T16	S60	V38	K61
T5	S16	Y30	S43			K86				L68		G6	-----	R48	R48	G18	K61	P39	R62

T6<br>A13'<br>G14'<br>A15'<br>G16'<br>A17'	L17<br>S18<br>A27<br>K28<br>S29<br>Y30<br>S32<br>R36<br>Q39<br>S43	S32<br>S33<br>I34<br>R36<br>L38<br>Q39<br>P42<br>S43<br>I44<br>Q45<br>F46<br>-----<br>N41'<br>R10<br>S16<br>L17<br>S18<br>E19<br>V26<br>A27<br>K28<br>S29<br>Y30<br>S32<br>S33<br>I34<br>R36<br>L38<br>Q39<br>P42<br>S43<br>I44<br>F46	-----<br>N41'<br>R10<br>S16<br>L17<br>S18<br>K28<br>S29<br>Y30<br>R36<br>Q39<br>P42<br>S43			G93<br>G94<br>G95<br>V96<br>N97<br>A98<br>S100<br>R101<br>Y102<br>E103<br>A107<br>Q108<br>P109<br>H110				G69<br>A70<br>T72<br>R73<br>M74<br>L75<br>D76<br>R77<br>R86<br>N89<br>R94<br>G95<br>V96<br>L97		T7<br>C8'<br>T9'<br>A10'<br>T7'	I11<br>Y12<br>S36<br>E37<br>R38<br>S47<br>R48<br>T50<br>R52<br>Q53<br>G69<br>T72	T50<br>R52<br>Q53<br>S56<br>R66<br>G69<br>R70<br>G71<br>T72<br>-----<br>I9<br>P10<br>I11<br>Y12<br>Y13<br>P35<br>S36<br>E37<br>R38<br>E39<br>I46<br>S47<br>R48<br>M49<br>T50<br>R52<br>Q53<br>V51<br>R66<br>G69<br>R70<br>G71<br>T72	R52<br>R66<br>G71<br>T72	T22<br>A6'<br>G7'<br>G8'<br>T9'<br>T10'	R62<br>G63<br>G65<br>-----<br>I4<br>S5<br>K27<br>R28<br>S29<br>S40<br>N43<br>Y44<br>R49<br>S60<br>K61<br>R62<br>G63	S40<br>Q41<br>N43<br>Y44<br>V45<br>T48<br>R49<br>E59<br>S60<br>K61<br>R62<br>G63<br>G64<br>G65<br>G66<br>Y67<br>I68<br>-----<br>K61'<br>N3<br>I4<br>S5<br>D6<br>I26<br>K27<br>R28<br>S29<br>E30<br>P39<br>S40<br>Q41<br>N43<br>Y44<br>T48<br>R49<br>E59<br>S60<br>K61<br>R62<br>G63	G63<br>G64<br>G65<br>G66<br>Y67<br>I68<br>-----<br>N3<br>I4<br>S5<br>K27<br>R28<br>S29<br>Q41<br>N43<br>Y44<br>R49<br>S60<br>K61<br>R62<br>G66<br>Y67<br>I68<br>K61'<br>G64'

																	G64<br>G65<br>G66<br>Y67<br>I68	
4HF1				3JR9				3JRA				3JRB				3JRC			
N	PC	4Å	HS	N	PC	4Å	HS	N	PC	4Å	HS	N	PC	4Å	HS	N	PC	4Å	HS
T6<br>C7<br>C8<br>T17'<br>G18'<br>T19'<br>G20'<br>T25'<br>-----<br>A6<br>C7<br>A8<br>T17'<br>G18'<br>T19'<br>G20'<br>T25'	R2<br>T4<br>L28<br>S38<br>S40<br>Y41<br>E43<br>Q44<br>S57<br>R59<br>Y65<br>-----<br>R2<br>T4<br>S5<br>L28<br>S38<br>S40<br>Y41<br>E43<br>Q44<br>R50<br>S57<br>R59<br>Y65	R2<br>T4<br>S5<br>K6<br>G7<br>V26<br>P27<br>L28<br>A29<br>I37<br>S38<br>L39<br>S40<br>Y41<br>E43<br>Q44<br>R50<br>S57<br>V58<br>R59<br>G64<br>Y65<br>D84<br>-----<br>R2<br>T4<br>S5<br>K6<br>Y9<br>V26<br>P27<br>L28<br>A29	R2<br>T4<br>K6<br>I37<br>S38<br>S40<br>Y41<br>E43<br>Q44<br>R50<br>S57<br>R59<br>Y65<br>-----<br>R2<br>T4<br>S5<br>K6<br>Y9<br>G36<br>I37<br>S38<br>S40<br>Y41<br>E43<br>Q44<br>R50<br>S57<br>R59<br>Y65	T5<br>T6<br>G7<br>T8<br>T16'<br>T17'<br>A18'<br>-----<br>T5'<br>T6'<br>G7'<br>T16<br>T17<br>G18<br>G20	N73<br>Q74<br>T75<br>N84<br>R85<br>R89<br>K90<br>-----<br>N73<br>Q74<br>T75<br>N84<br>R85<br>T87<br>R89<br>K90	G72<br>N73<br>Q74<br>T75<br>R76<br>I83<br>N84<br>R85<br>T87<br>R89<br>K90<br>-----<br>G72<br>N73<br>Q74<br>T75<br>R76<br>I83<br>N84<br>R85<br>T87<br>R89<br>K90	G72<br>N73<br>Q74<br>T75<br>R85<br>R89<br>-----<br>G72<br>N73<br>Q74<br>T75<br>R85<br>R89	T5<br>T6<br>G7<br>A16'<br>T17'<br>G18'<br>-----<br>T5'<br>T6'<br>A7'<br>T16<br>T17<br>A18	N73<br>Q74<br>T75<br>N84<br>R85<br>T87<br>R89<br>K90<br>-----<br>N73<br>Q74<br>T75<br>N84<br>R85<br>T87<br>R89<br>K90<br>K91	G72<br>N73<br>Q74<br>T75<br>R76<br>I83<br>N84<br>R85<br>T87<br>L88<br>R89<br>K90<br>-----<br>E59<br>G72<br>N73<br>Q74<br>T75<br>R76<br>I83<br>N84<br>R85<br>T87<br>R89<br>K90<br>K91	G72<br>N73<br>Q74<br>T75<br>R85<br>R89<br>-----<br>G72<br>N73<br>Q74<br>T75<br>R85<br>R89	T5<br>T6<br>G7<br>T8<br>C17'<br>A18'<br>-----<br>T5'<br>T6'<br>G7'<br>T16<br>C17<br>A18<br>G20	N73<br>Q74<br>T75<br>N84<br>R85<br>T87<br>R89<br>-----<br>N73<br>Q74<br>T75<br>N84<br>R85<br>T87<br>R89<br>K90	G72<br>N73<br>Q74<br>T75<br>R76<br>I83<br>N84<br>R85<br>T87<br>L88<br>R89<br>-----<br>G72<br>N73<br>Q74<br>T75<br>R76<br>I83<br>N84<br>R85<br>T87<br>L88<br>R89<br>K90<br>N98	G72<br>N73<br>Q74<br>T75<br>R85<br>R89<br>-----<br>G72<br>N73<br>Q74<br>T75<br>R85<br>R89	T6<br>G7<br>C16'<br>C17'<br>A18'<br>-----<br>T5'<br>T6'<br>G7'<br>C16<br>T17<br>G18	N73<br>Q74<br>T75<br>N84<br>R85<br>T87<br>R89<br>K90<br>-----<br>N73<br>Q74<br>T75<br>N84<br>R85<br>T87<br>R89<br>K90	G72<br>N73<br>Q74<br>T75<br>R76<br>I83<br>N84<br>R85<br>T87<br>R89<br>K90<br>-----<br>M67<br>G72<br>N73<br>Q74<br>T75<br>R76<br>I83<br>N84<br>R85<br>T87<br>R89<br>K90	G72<br>N73<br>Q74<br>T75<br>R85<br>R89<br>-----<br>G72<br>N73<br>Q74<br>T75<br>R85<br>R89

		G36<br>I37<br>S38<br>L39<br>S40<br>Y41<br>E43<br>Q44<br>R50<br>S57<br>V58<br>R59<br>G64<br>Y65<br>D84																	
3JRD				3JRE				3JRF				3JRG				3JRH			
N	PC	4Å	HS	N	PC	4Å	HS	N	PC	4Å	HS	N	PC	4Å	HS	N	PC	4Å	HS
T5<br>T6<br>G7<br>A16'<br>C17'<br>A18'<br>-----<br>T5'<br>T6'<br>G7'<br>A16<br>T17<br>G18<br>G20	N73<br>Q74<br>T75<br>N84<br>R85<br>T87<br>R89<br>K90<br>-----<br>N73<br>Q74<br>T75<br>N84<br>R85<br>T87<br>R89<br>K90<br>K91	G72<br>N73<br>Q74<br>T75<br>R76<br>I83<br>N84<br>R85<br>T87<br>R89<br>K90<br>-----<br>E59<br>G72<br>N73<br>Q74<br>T75<br>R76<br>I83<br>N84<br>R85<br>T87<br>R89	G72<br>N73<br>Q74<br>T75<br>R85<br>R89<br>-----<br>G72<br>N73<br>Q74<br>T75<br>R85<br>R89	T5<br>T6<br>G7<br>T16'<br>C17'<br>A18'<br>-----<br>T5'<br>T6'<br>G7'<br>A16<br>T17<br>G18<br>G20	N73<br>Q74<br>T75<br>N84<br>R85<br>T87<br>R89<br>K90<br>-----<br>N73<br>Q74<br>T75<br>N84<br>R85<br>T87<br>R89<br>K90	G72<br>N73<br>Q74<br>T75<br>R76<br>I83<br>N84<br>R85<br>T87<br>L88<br>R89<br>K90<br>-----<br>G72<br>N73<br>Q74<br>T75<br>R76<br>I83<br>N84<br>R85<br>T87<br>R89	G72<br>N73<br>Q74<br>T75<br>R85<br>R89<br>-----<br>G72<br>N73<br>Q74<br>T75<br>R85<br>R89	T5<br>T6<br>G7<br>T16'<br>C17'<br>-----<br>T5'<br>T6'<br>G7'<br>T17<br>G18<br>G20	N73<br>Q74<br>T75<br>N84<br>R85<br>R89<br>K90<br>-----<br>N73<br>Q74<br>T75<br>N84<br>R85<br>T87<br>R89	G72<br>N73<br>Q74<br>T75<br>R76<br>I83<br>N84<br>R85<br>T87<br>R89<br>K90<br>-----<br>G72<br>N73<br>Q74<br>T75<br>R76<br>I83<br>N84<br>R85<br>T87<br>R89	G72<br>N73<br>Q74<br>T75<br>R85<br>R89<br>-----<br>G72<br>N73<br>Q74<br>T75<br>R85<br>R89	T5<br>T6<br>G7<br>T8<br>T16'<br>C17'<br>C18'<br>-----<br>T5'<br>T6'<br>G7'<br>T16<br>T17<br>G20	N73<br>Q74<br>T75<br>N84<br>R85<br>T87<br>R89<br>K90<br>-----<br>N73<br>Q74<br>T75<br>N84<br>R85<br>T87<br>R89<br>K90<br>K91	G72<br>N73<br>Q74<br>T75<br>R76<br>I83<br>N84<br>R85<br>T87<br>L88<br>R89<br>K90<br>-----<br>E59<br>G72<br>N73<br>Q74<br>T75<br>R76<br>I83<br>N84<br>R85<br>T87	G72<br>N73<br>Q74<br>T75<br>R85<br>R89<br>-----<br>G72<br>N73<br>Q74<br>T75<br>R85<br>R89	T5<br>T6<br>G7<br>T16'<br>G17'<br>A18'<br>-----<br>T5'<br>T6'<br>G7'<br>T15<br>G17<br>G18	N73<br>Q74<br>T75<br>N84<br>R85<br>T87<br>R89<br>K90<br>-----<br>N73<br>Q74<br>T75<br>N84<br>R85<br>T87<br>R89<br>K90	G72<br>N73<br>Q74<br>T75<br>R76<br>I83<br>N84<br>R85<br>G86<br>T87<br>R89<br>K90<br>-----<br>G72<br>N73<br>Q74<br>T75<br>R76<br>I83<br>N84<br>R85<br>T87<br>G86	G72<br>N73<br>Q74<br>T75<br>R85<br>R89<br>-----<br>G72<br>N73<br>Q74<br>T75<br>R85<br>R89

		K90<br>K91				K90								R89<br>K90<br>K91				R89<br>K90	
3JRI				5E3O				5E3N				5E3M				5E3L			
N	PC	4Å	HS	N	PC	4Å	HS	N	PC	4Å	HS	N	PC	4Å	HS	N	PC	4Å	HS
T5<br>T6<br>G7<br>A17'<br>-----<br>T5'<br>T6'<br>G7'<br>T16<br>G17	N73<br>Q74<br>T75<br>N84<br>R85<br>T87<br>R89<br>-----<br>N73<br>Q74<br>T75<br>N84<br>R85<br>T87<br>R89<br>K90<br>K91	G72<br>N73<br>Q74<br>T75<br>R76<br>I83<br>N84<br>R85<br>L88<br>T87<br>R89<br>-----<br>E59<br>G72<br>N73<br>Q74<br>T75<br>R76<br>I83<br>N84<br>R85<br>T87<br>R89<br>K90<br>K91	G72<br>N73<br>Q74<br>T75<br>R85<br>R89<br>------<br>G72<br>N73<br>Q74<br>T75<br>R85<br>R89	T5<br>T6<br>G7<br>G8<br>T16'<br>C17'<br>-----<br>T5'<br>T6'<br>G7'<br>G8'<br>T16<br>T17	N73<br>Q74<br>T75<br>N84<br>R85<br>T87<br>R89<br>K90<br>-----<br>N73<br>Q74<br>T75<br>N84<br>R85<br>T87<br>R89<br>K90	G72<br>N73<br>Q74<br>T75<br>R76<br>I83<br>N84<br>R85<br>G86<br>T87<br>R89<br>K90<br>-----<br>G72<br>N73<br>Q74<br>T75<br>R76<br>I83<br>N84<br>R85<br>T87<br>R89<br>K90	G72<br>N73<br>Q74<br>T75<br>R85<br>-----<br>G72<br>N73<br>Q74<br>T75<br>R85<br>R89	T5<br>T6<br>G7<br>T8<br>T16'<br>C17'<br>-----<br>T5'<br>T6'<br>G7'<br>T16<br>T17	N73<br>Q74<br>T75<br>N84<br>R85<br>T87<br>R89<br>K90<br>-----<br>N73<br>Q74<br>T75<br>N84<br>R85<br>T87<br>R89<br>K90	G72<br>N73<br>Q74<br>T75<br>R76<br>I83<br>N84<br>R85<br>T87<br>R89<br>K90<br>-----<br>G72<br>N73<br>Q74<br>T75<br>R76<br>I83<br>N84<br>R85<br>T87<br>R89<br>K90	G72<br>N73<br>Q74<br>T75<br>R85<br>R89<br>-----<br>G72<br>N73<br>Q74<br>T75<br>R85<br>R89	T5<br>A6<br>G7<br>T8<br>T16'<br>C17'<br>A18'<br>-----<br>T5'<br>A6'<br>G7'<br>T16<br>C17<br>G18<br>G20	N73<br>Q74<br>T75<br>N84<br>R85<br>T87<br>R89<br>K90<br>-----<br>Q74<br>T75<br>R76<br>N84<br>R85<br>T87<br>R89<br>K90	G72<br>N73<br>Q74<br>T75<br>R76<br>I83<br>N84<br>R85<br>T87<br>R89<br>K90<br>-----<br>G72<br>N73<br>Q74<br>T75<br>R76<br>I83<br>N84<br>R85<br>T87<br>R89<br>K90	G72<br>N73<br>Q74<br>T75<br>R85<br>R89<br>-----<br>G72<br>N73<br>Q74<br>T75<br>R85<br>R89	T5<br>G6<br>G7<br>T8<br>T16'<br>C17'<br>A18'<br>-----<br>T5'<br>G6'<br>G7'<br>T16<br>C17<br>G18	N73<br>Q74<br>T75<br>N84<br>R85<br>T87<br>R89<br>K90<br>-----<br>N73<br>Q74<br>T75<br>N84<br>R85<br>T87<br>R89<br>K90	G72<br>N73<br>Q74<br>T75<br>R76<br>I83<br>N84<br>R85<br>T87<br>R89<br>K90<br>-----<br>G72<br>N73<br>Q74<br>T75<br>R76<br>I83<br>N84<br>R85<br>T87<br>R89<br>K90	G72<br>N73<br>Q74<br>T75<br>R85<br>R89<br>-----<br>G72<br>N73<br>Q74<br>T75<br>R85<br>R89
5DTD				5DS9				4IHV											
N	PC	4Å	HS	N	PC	4Å	HS	N	PC	4Å	HS								
T5<br>C6<br>G7<br>T8<br>T16'<br>C17'<br>A18'<br>-----	N73<br>Q74<br>T75<br>N84<br>R85<br>T87<br>R89<br>K90	G72<br>N73<br>Q74<br>T75<br>R76<br>I83<br>N84<br>R85	G72<br>N73<br>Q74<br>T75<br>R85<br>R89<br>-----<br>G72	T5<br>A6<br>G7<br>T8<br>T16'<br>C17'<br>A18'<br>-----	N73<br>Q74<br>T75<br>N84<br>R85<br>T87<br>R89<br>K90	G72<br>N73<br>Q74<br>T75<br>R76<br>I83<br>N84<br>R85	G72<br>N73<br>Q74<br>T75<br>R85<br>R89<br>-----<br>G72	T5<br>T6<br>G7<br>T8<br>T16'<br>C17'<br>A18'<br>-----	N73<br>Q74<br>T75<br>N84<br>R85<br>T87<br>R89<br>K90	G72<br>N73<br>Q74<br>T75<br>R76<br>I83<br>N84<br>R85	G72<br>N73<br>Q74<br>T75<br>R85<br>R89<br>-----<br>G72								

T5'<br>C6'<br>G7'<br>T16<br>T17<br>G18<br>G20	-----<br>N73<br>Q74<br>T75<br>N84<br>R85<br>T87<br>R89<br>K90	T87<br>R89<br>K90<br>-----<br>G72<br>N73<br>Q74<br>T75<br>R76<br>I83<br>N84<br>R85<br>T87<br>R89<br>K90	N73<br>Q74<br>T75<br>R85<br>R89	T5'<br>A6'<br>G7'<br>T16<br>T17<br>G18	-----<br>N73<br>Q74<br>T75<br>N84<br>R85<br>T87<br>R89<br>K90	T87<br>R89<br>K90<br>-----<br>G72<br>N73<br>Q74<br>T75<br>R76<br>I83<br>N84<br>R85<br>T87<br>R89<br>K90	N73<br>Q74<br>T75<br>R85<br>R89	T5'<br>T6'<br>G7'<br>T16<br>T17<br>G18	-----<br>N73<br>Q74<br>T75<br>N84<br>R85<br>T87<br>R89<br>K90	L88<br>T87<br>R89<br>K90<br>-----<br>G72<br>N73<br>Q74<br>T75<br>R76<br>I83<br>N84<br>R85<br>T87<br>R89<br>K90	N73<br>Q74<br>T75<br>R85<br>R89								

Table S5. Data from literature (ΔG_b) and calculated data (other parameters) for the set of HTH-type proteins complexed with DNA duplexes that were used to find correlations: changes in Gibbs free energy during binding (ΔG_b) and characteristics of interfaces: number of polar contacts (PC), characteristics of amino acids within 4 Å vicinity of PC, amino acids participated in polar contacts (AA in PC) and amino acids within 4 Å vicinity of hot spots (HS) that are nucleotides with ≥3 PC. The characteristics are number of amino acids (AA), mean length of sidechain (SC) in AA, total atoms in AA, sum of positively charged and aromatic amino acids (F, Y, W, H, R, K). Hydrogen atoms were not counted. Results for alternative conformations were averaged; the values refer to 1 domain. Parameter C_y' was calculated using equation (5), mean length of SC and number of amino acids within 4 Å vicinity of PC; the values used for linearization are colored. The efficiency of the complexes was calculated using equation (8); low efficient complexes (efficiency < 55%) are colored with red.

PDB id	$-\Delta G_b$, kJ/mol	Number of polar contacts	Mean length of SC of AA in PC	Total atoms in AA in PC	Number of AA in PC	Mean length of SC	Total atoms in AA	Number of AA in 4Å vicinity	Mean length of SC of AA in HS	Total atoms in HS vicinity	Number of AA in HS vicinity	C_y'	Efficiency, %
4Z58	50	15	4.10	41	10	4.00	84	21	3.50	28	8	439	55.4
4R24	45.3	20	4.92	59	12	4.38	105	24	4.91	54	11	591	44.7
4WLW	44.6	21	5.80	58	10	4.47	85	19	5.25	21	4	484	47.7
3IV5	51.4	14.5	4.76	40.5	8.5	4.48	56	12.5	4.33	26	6	290	64.6
5D8C	43.3	15	5.86	41	7	4.38	85.5	19.5	4.67	28	6	446	47.7
3ZKC	37.5	17	3.87	44.5	11.5	4.09	92	22.5	4.16	52	12.5	497	39.7
3O9X	48.2	18	4.89	44	9	3.93	106	27	5.50	11	2	581	48.0
5H3R	51.3	16	5.10	51	10	3.59	97	27	4.22	38	9	515	53.5
4WWC	60.2	20	4.50	54	12	4.38	98.5	22.5	4.75	38	8	554	61.0
3H0D	44.4	23	4.10	59.5	14.5	3.89	111	28.5	4.14	76.5	18.5	647	42.2
4HF1	43.9	18.5	4.79	57.5	12	3.96	93	23.5	4.83	70	14.5	513	45.9
3IV5	51.4	14.5	4.76	40.5	8.5	4.48	56	12.5	4.33	26	6	290	64.6
3JR9	51.4	14	4.87	36.5	7.5	4.45	49	11	4.33	26	6	251	66.9
3JRA	42.9	14.5	4.76	40.5	8.5	4.48	56	12.5	4.33	26	6	290	53.9
3JRB	49.3	14.5	4.73	35.5	7.5	4.38	52.5	12	4.33	26	6	272	63.0

3JRC	36.3	14.5	4.75	38	8	4.43	51	11.5	4.33	26	6	264	46.7
3JRD	47.7	15	4.76	40.5	8.5	4.50	54	12	4.33	26	6	282	60.3
3JRE	49.3	14	4.75	38	8	4.43	51	11.5	4.33	26	6	261	63.6
3JRF	48.9	11.5	4.86	34	7	4.43	46.5	10.5	4.33	26	6	226	65.2
3JRG	46.1	13.5	4.76	40.5	8.5	4.48	56	12.5	4.33	26	6	284	58.2
3JRH	39.2	13	4.75	38	8	4.08	49	12	4.33	26	6	246	51.3
3JRI	39.7	13	4.75	38	8	4.46	53.5	12	4.33	26	6	269	50.8
4IHV	40.1	14	4.75	38	8	4.43	51	11.5	4.33	26	6	261	51.7
5E3O	40.1	12.5	4.75	38	8	4.26	49	11.5	4.09	22.5	5.5	243	52.6
5E3N	40.7	13	4.75	38	8	4.45	49	11	4.33	26	6	246	53.2
5E3M	41.5	16	4.75	38	8	4.45	49	11	4.33	26	6	260	53.6
5E3L	39.9	15	4.75	38	8	4.45	49	11	4.33	26	6	256	51.7
5DTD	46	14.5	4.75	38	8	4.45	49	11	4.33	26	6	254	59.7
5DS9	48.5	15.5	4.75	38	8	4.45	49	11	4.33	26	6	258	62.7

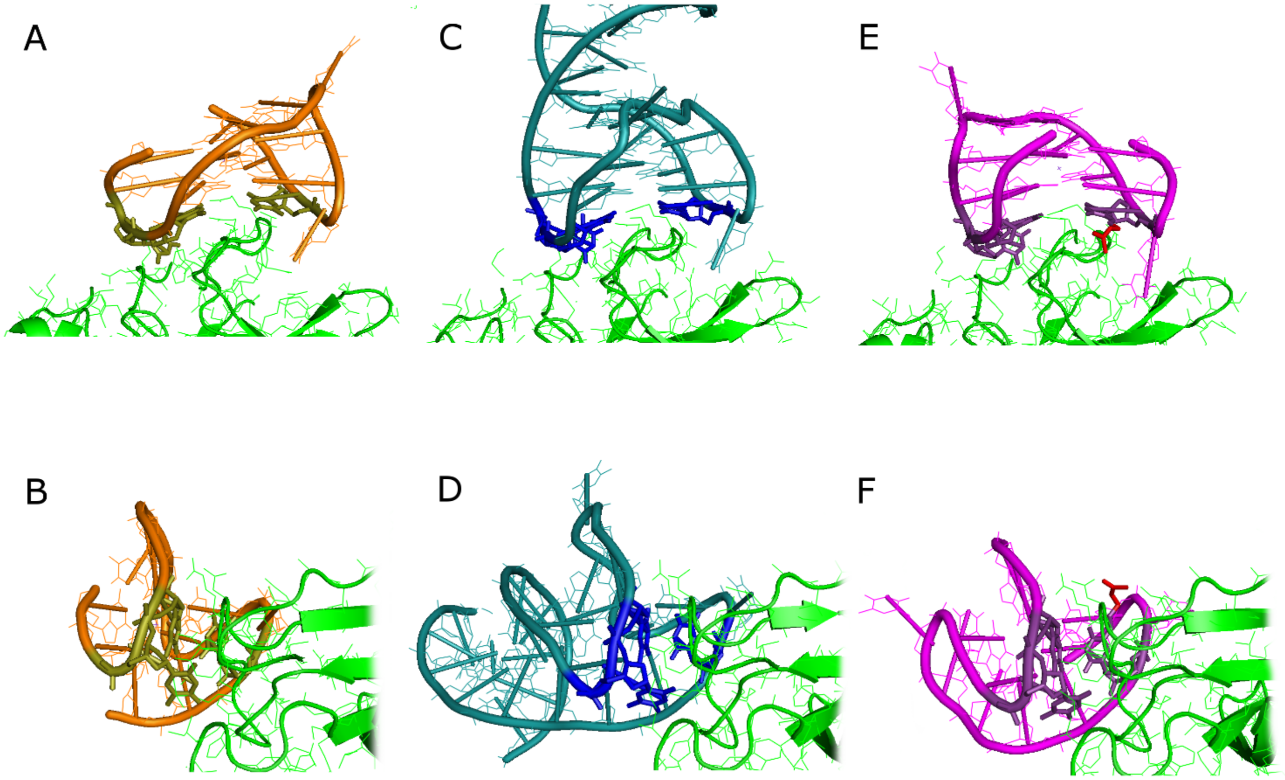

Figure S1. Thrombin complexes with DNA aptamers: HD1 (A,B), RE31 (C,D), and T4K (E,F) in two different views: front view (A,C, and E) and side view (B,D, and F). Thrombin is colored in green. The substituent in thymine (T4) is colored in red. The positioning of recognizing loops is common in all three complexes.

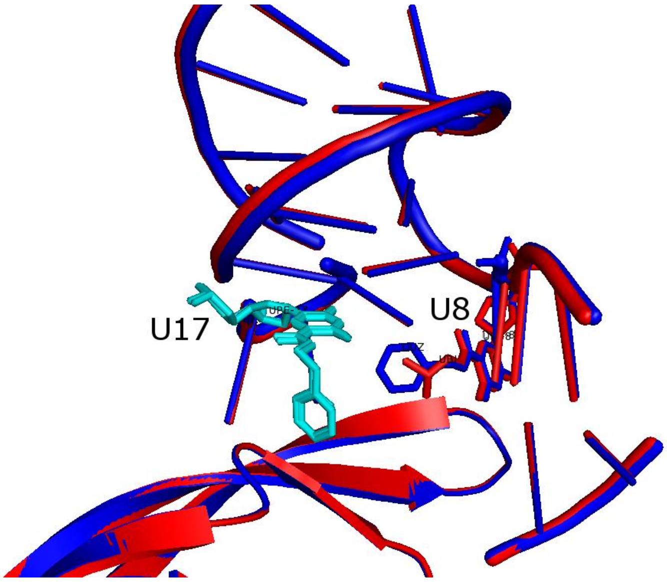

Figure S2. PDGFB complexes with modified DNA aptamers SL4 (in red color) and SL5 (in blue color) aligned together. 'Hot spot' residue (U17, benzyl modified uridine; in light blue) and the sole differing residue (U8, i-butyl or benzyl modified uridine) are shown in details. U17 is in contact with benzyl substituent but not with i-butyl substituent.
